# The CK1δ/ε-AES axis regulates tumorigenesis and metastasis in colorectal cancer

**DOI:** 10.7150/thno.53901

**Published:** 2021-03-04

**Authors:** Zhongyuan Wang, Liang Zhou, Yejun Wang, Quanzhou Peng, Huan Li, Xin Zhang, Zijie Su, Jiaxing Song, Qi Sun, Sapna Sayed, Shanshan Liu, Desheng Lu

**Affiliations:** 1 Guangdong Provincial Key Laboratory of Regional Immunity and Diseases, Cancer Research Center, Department of Pharmacology, Shenzhen University Health Science Center, Shenzhen 518055, China; 2 Department of Physiology, School of Medicine, Health Science Center, Shenzhen University, Shenzhen 518060, China; 3 Department of Pathology, Shenzhen People's Hospital, the Second Clinical Medical College of Jinan University, Shenzhen 518020, China

**Keywords:** AES, CK1δ/ε, SKP2, colorectal cancer, Wnt/β-catenin signaling

## Abstract

**Background:** Amino-terminal enhancer of split (AES) has been identified as a tumor and metastasis suppressor in some cancers including colorectal cancer (CRC), but very little is known about the regulation of AES expression.

**Methods:** Bioinformatics analysis was used to investigate the expression patterns of AES, CK1δ and CK1ε. The co-immunoprecipitation, GST pull-down, Western Blot, real-time PCR and immunohistochemistry were performed to study the mechanism underlying the regulation of AES expression by CK1δ/ε. The biological function was assessed by *in vitro* colony formation, transwell, sphere formation, tumor organoids, *in vivo* tumor metastasis model and patient-derived colorectal tumor xenografts (PDTX) model.

**Results:** A strong inverse relationship was observed between the expression of AES and the expression of CK1δ/ε. Mechanically, AES could interact with CK1δ/ε and SKP2 using its Q domain. SKP2 mediated the ubiquitination and degradation of AES in a CK1δ/ε-dependent manner. CK1δ/ε phosphorylated AES at Ser121 and accelerated the SKP2-mediated ubiquitination and degradation of AES. In colon cancer cells, CK1δ/ε antagonized the effect of wild-type AES but not that of its mutant (S121A) on Wnt and Notch signaling, leading to an increase in the expression of Wnt target genes and Notch target genes. By downregulating the expression of AES, CK1δ/ε enhanced anchorage-independent growth, migration, invasion and sphere formation in colon cancer cells. CK1δ/ε also promoted the growth of APC^min/+^ colorectal tumor organoids and liver metastasis in colon cancer mouse models through the regulation of AES degradation. Furthermore, CK1 inhibitor SR3029 treatment suppressed tumor growth via stabilizing AES in APC^min/+^ colorectal tumor organoids and patient-derived colorectal tumor xenografts (PDTX).

**Conclusions:** Our results revealed that the CK1δ/ε-AES axis is important for CRC tumorigenesis and metastasis, and targeted inhibition of this axis may be a potential therapeutic strategy for CRC.

## Introduction

Colorectal cancer (CRC) is the third most commonly diagnosed malignancy worldwide, associated with a high rate of mortality 1. The distant metastasis is a major cause of cancer-related death in CRC patients. The liver is the most common site of metastatic dissemination and about a quarter of patients with CRC had liver metastases at primary diagnosis. Nearly half of patients will ultimately develop liver metastases during their lifetime 2, 3. A better understanding of the molecular mechanisms underlying CRC metastasis is imperative to develop innovative therapies against this malignancy.

The CRC metastatic cascade is a complex, multistep biological process involving local invasion, intravasation, survival in the circulation, extravasation and colonization in the distant site 4-7. Multiple signaling pathways have been implicated in CRC metastasis formation, such as Wnt/β-catenin, Notch and TGF-β 8-10. Amino-terminal enhancer of split (AES), a member of the Groucho/transducin-like enhancer of split/Grorelated gene (Gro/TLE/Grg) family of transcriptional co-repressors, has been identified as a metastasis suppressor for CRC 11. Previous studies showed that AES could inhibit several signaling pathways, including the Notch pathway, the Wnt/β-catenin pathway, the Androgen Receptor (AR) and Bone Morphogenetic Protein (BMP) signaling pathways 11-14. These studies indicate that AES may exert its suppressive effect on CRC metastasis through mediating the multiple pathways. Maintaining or enhancing the metastasis-suppressive effect of AES might be useful for the prevention and treatment of CRC metastasis. However, little is known about the regulation of AES expression during the process of CRC metastasis.

The human casein kinase 1 (CK1) is a family of serine/threonine (Ser/Thr) protein kinases which consists of six isoforms (α, δ, ε, γ1, γ2 and γ3), with CK1δ and CK1ε sharing a highly homologous sequence with 98% identity in their kinase domain 15, 16. CK1 is evolutionarily conserved in eukaryotes with a conserved core kinase domain and a variable small N-terminal lobe and a variable large C-terminal lobe, which are involved in regulation of subcellular localization, substrate specificity and kinase activity of CK1 15. CK1 phosphorylates a variety of substrates, and modulates diverse cellular processes such as the Wnt signaling pathway, circadian rhythms, membrane trafficking, cytoskeleton maintenance, DNA damage response, ribosome biogenesis, cell cycle and differentiation, and immune response 15, 17-19. CK1 could phosphorylate p53 and its negative regulator MDM2 to influence p53 degradation by MDM2 20, 21. In the Wnt pathway, CK1 phosphorylates several critical components and both positively and negatively regulates Wnt signaling 16. In the destruction complex, CK1α phosphorylates β-catenin and initiates β-catenin degradation by proteasome 22. CK1δ and CK1ε are also able to phosphorylate adenomatosis polyposis protein (APC) and scaffolding protein Axin to facilitate β-catenin degradation 23. Upon Wnt stimulation, CK1 phosphorylates low-density lipoprotein receptor-related protein 6 (LRP6) and dishevelled (DVL) at multiple sites, which is an essential step in promoting Wnt signaling 24-26. CK1δ and CK1ε have been reported to modulate colorectal, breast and pancreatic cancer progression through regulating Wnt and Hedgehog (Hh) signaling 27-29. A recent study showed that CK1δ or CK1ε is specifically upregulated in intestinal stem cells (ISCs) and is essential for ISC maintenance 30.

S phase kinase-associated protein 2 (SKP2) is an essential component of the SCF (Skp1-Cullin-1-F-box) ubiquitin E3 ligase complex. SKP2 is responsible for substrate recognition of the SCF E3 ligase complex 31. Several substrates of SKP2 have been identified, including p27, Akt, c-Myc and LKB1 32-35. Overexpression of SKP2 has been detected in CRC tissues and cell lines 36. Increasing evidence indicates that SKP2 can function as an oncogene crucial for CRC development and metastasis 36, 37.

In the present study, we found that the expression of AES was regulated by CK1δ/ε. CK1δ/ε enhanced the SKP2-mediated ubiquitination and degradation of AES through phosphorylating AES at Ser121. In colon cancer cells, CK1δ/ε-induced wild-type AES degradation led to the activation of the Wnt and Notch signaling pathways, thereby increasing the anchorage-independent growth, migration, invasion, and sphere formation of colon cancer cells, promoting tumor growth and liver metastasis in CRC mouse models, while CK1δ/ε had little effect on AES mutant (S121A)-mediated activities. Importantly, a CK1 inhibitor SR3029 treatment suppressed tumor growth via stabilizing AES in APC^min/+^ colorectal tumor organoids and PDTX model.

## Materials and Methods

### RNA isolation and real-time PCR analyses

Total RNA was isolated by RNAiso Plus (TaKaRa) according to the manufacturer's instructions. First strand cDNA was synthesized using 1 μg total RNA in each cDNA synthesis reaction by the Primescript RT Reagent Kit (TaKaRa). Then prepared cDNA was subjected to real-time PCR analysis using ABI Prism 7500 Real-Time PCR System with Eastep qPCR Master Mix (Promega) and primer mixtures (primer sequences are shown in Table S1). The comparative Ct method was used to analyze relative expression of genes. The data were collected from three to five independent experiments and each sample was analyzed in three technical replicates.

### Immunoprecipitation and immunoblot

For immunoprecipitation, cells were lysed with buffer containing 20 mM Tris-HCl, pH7.4, 150 mM NaCl, 1 mM EDTA, 1 mM EGTA, 1% Triton X-100 supplemented with phosphatase-inhibitor cocktail, protease-inhibitor cocktail and 1 mM PMSF followed by mild sonication. After centrifugation at 12,000 rpm for 15 min, the supernatant fractions were subjected to immunoprecipitation using the anti-Flag M2 affinity gel or anti-V5 affinity gel or the indicated primary antibody and protein A/G agarose beads at 4 °C. For λ-phosphatase treatment, cells were lysed with buffer containing 20 mM Tris-HCl, pH7.4, 150 mM NaCl, 1 mM MnCl_2_, 1% Triton X-100 supplemented with protease-inhibitor cocktail and 1 mM PMSF followed by mild sonication. After centrifugation at 12,000 rpm for 15 min, the supernatant fractions were incubated with in the presence or absence of λ-phosphatase for 30 min at 30 °C and then subjected to immunoprecipitation at 4 °C. For immunoblot, cells or tumor tissues were lysed in RIPA buffer (50 mM Tris-HCl, pH7.4, 150 mM NaCl, 1% NP-40, 0.1% SDS) supplemented with phosphatase-inhibitor cocktail, protease-inhibitor cocktail and 1 mM PMSF followed by mild sonication. Equal proteins were separated by SDS-PAGE and transferred to PVDF membranes. Immunoblot was carried out with primary antibodies (listed in Table S2) and horseradish peroxidase-conjugated secondary antibody. The membrane was then incubated with ECL Plus Western Blotting Substrate and detected using either X-ray film or Chemiluminescent Imaging System (Tanon 5200, Shanghai, China).

### Organoid culture

The colon of B6-APC^min/+^ mice was collected and opened longitudinally. Then tumor regions were identified and dissected out. After washing with cold PBS, the pieces were incubated in 2 mM EDTA/PBS for 60 min on ice to remove normal epithelial cells, and then tumor crypts were isolated in digestion buffer (2.5% FBS, 200 U/ml type IV collagenase, 125 μg/ml type II dispase in DMEM) for 1 h at 37 °C and purified by successive centrifugation steps. Tumor crypts were resuspended in 5 mg/ml Matrigel on ice and 50 μl Matrigel was plated in each well of pre-warmed 24-well plates. After polymerization for 15min at 37°C, 500μl advanced DMEM/F12 containing 50 ng/ml EGF, 50 ng/ml Noggin, 500 ng/ml R-Spondin1, 100 ng/ml Wnt3A, 10 mM nicotinamide, 1mM N-acetylcysteine, and 10 μM Y-27631 was added and refreshed every two to three days.

### Immunofluorescence

Immunofluorescence staining was performed as previously described 38. Briefly, cells grown on coverslips or the sectioned organoids (7 μm) were fixed with 4% paraformaldehyde and permeabilized with 0.4% Triton X-100. Following blocking with 10% goat serum, the samples were incubated with the indicated primary antibodies in blocking buffer (10% goat serum in PBS) overnight at 4 °C, and then rinsed and incubated with secondary Alexa Fluor 488-conjugated anti-mouse antibody and Alexa Fluor 594-conjugated anti-rabbit antibody (Life Technologies) for 1 h at room temperature. Cells were then rinsed with PBS, stained with 4,6-diamidino-2-phenylindole (DAPI) and mounted. The slides were observed with a fluorescence microscope (LSM880, ZEISS) at Instrumental Analysis Center of Shenzhen University.

### Histology and immunohistochemistry analyses

Samples were fixed in 4% formaldehyde in PBS for 24 h, then dehydrated and embedded in paraffin, sectioned at 6 μm before H&E staining and immunohistochemistry were performed. The primary antibodies used for immunohistochemistry are listed in Supplementary Table 2. The score of immunoreactivity was evaluated according to the method previously described 39.

### Animal model studies

All animal experimental protocols were approved by the Animal Research Ethics Committee of Shenzhen University (permit number: AEWC-201412003). BALB/c nude mice and NPG mice were obtained from Beijing Vital River Laboratory Animal Technology Co., Ltd., Beijing, China. B6-APC^min/+^ mice were purchased from Nanjing Biomedical Research Institute of Nanjing University, Nanjing, China. All the mice were maintained in a specific pathogen free facility with six mice per cage in the animal research center of Shenzhen University.

For *in vivo* metastasis assay, twenty-four 8-week-old male BALB/c nude mice were randomly divided into seven groups and intrasplenicly injected with 1 × 10^6^ the indicated lentivirus infected HCT116 cells. One month after implantation, mice were killed, and livers were dissected and photographed.

For patient-derived colorectal tumor xenografts (PDTX), human CRC specimens were collected from two patients who had not been previously treated with radiotherapy and chemotherapy in compliance with the Human Research Ethics Committee of Shenzhen University (permit number: 201619008). A written consent form was signed by the patient prior to participation in the study. Patients were diagnosed with CRC by endoscopic biopsies and subsequently confirmed by histology in the First Affiliated Hospital of Shenzhen University. Establishment of PDTXs was performed as previous described 38, 40. Briefly, necrotic and supporting tissues of patient's tumor were removed and about 20-30 mg of tumor fragments were subcutaneously (s.c.) implanted into the right flank of 8-week-old male NPG mice using a trocar. Successfully engrafted tumor models were then passaged. After successful expansion of the F1-F3 generations of two patient-derived colorectal tumor specimens in NPG mice, F3 PDTX fragments (about 1 mm^3^) were implanted s.c. into the right flank of 8-week-old male NPG mice. Tumor growth was measured every 3 days. When the tumors reached about 50 mm^3^, mice were randomly divided into two groups and intraperitoneal (i.p.) injected with the vehicle (0.8% DMSO/12% Cremophor/8% ethanol in saline) or 5 mg/kg SR3029 in vehicle every 3 days. Tumor sizes were measured with a caliper and tumor volumes were calculated using the formula: 0.5 × length × width^2^. One month after treatment, mice were sacrificed, and tumors were collected and photographed.

### Gene expression analyses

Gene expression data for 473 colon adenocarcinomas and 41 non-malignant adjacent colon tissues were downloaded from The Cancer Genome Atlas (TCGA; https://portal.gdc.cancer.gov/) and 110 colon adenocarcinomas and 34 non-malignant adjacent colon tissues were downloaded from GEO database (GSE20916). The expression levels of target genes were extracted and measured as Fragments Per Kilobase per Million pairs of reads (FPKMs). Gene expression was compared between tumor and adjacent normal tissues, and Wilcoxon rank sum tests with continuity correction were performed. A cutoff value of 0.05 was preset as significance level.

### Statistical analyses

Statistical analyses were carried out with GraphPad Prism8.00 (GraphPad Software). The data were analyzed by Student's t test or Two-way ANOVA. Results are presented as mean ± SD. Statistical significance was set to P < 0.05.

## Results

### The expression pattern of AES is inversely correlated with the expression of CK1δ and CK1ε in CRC

**Figure 1:**
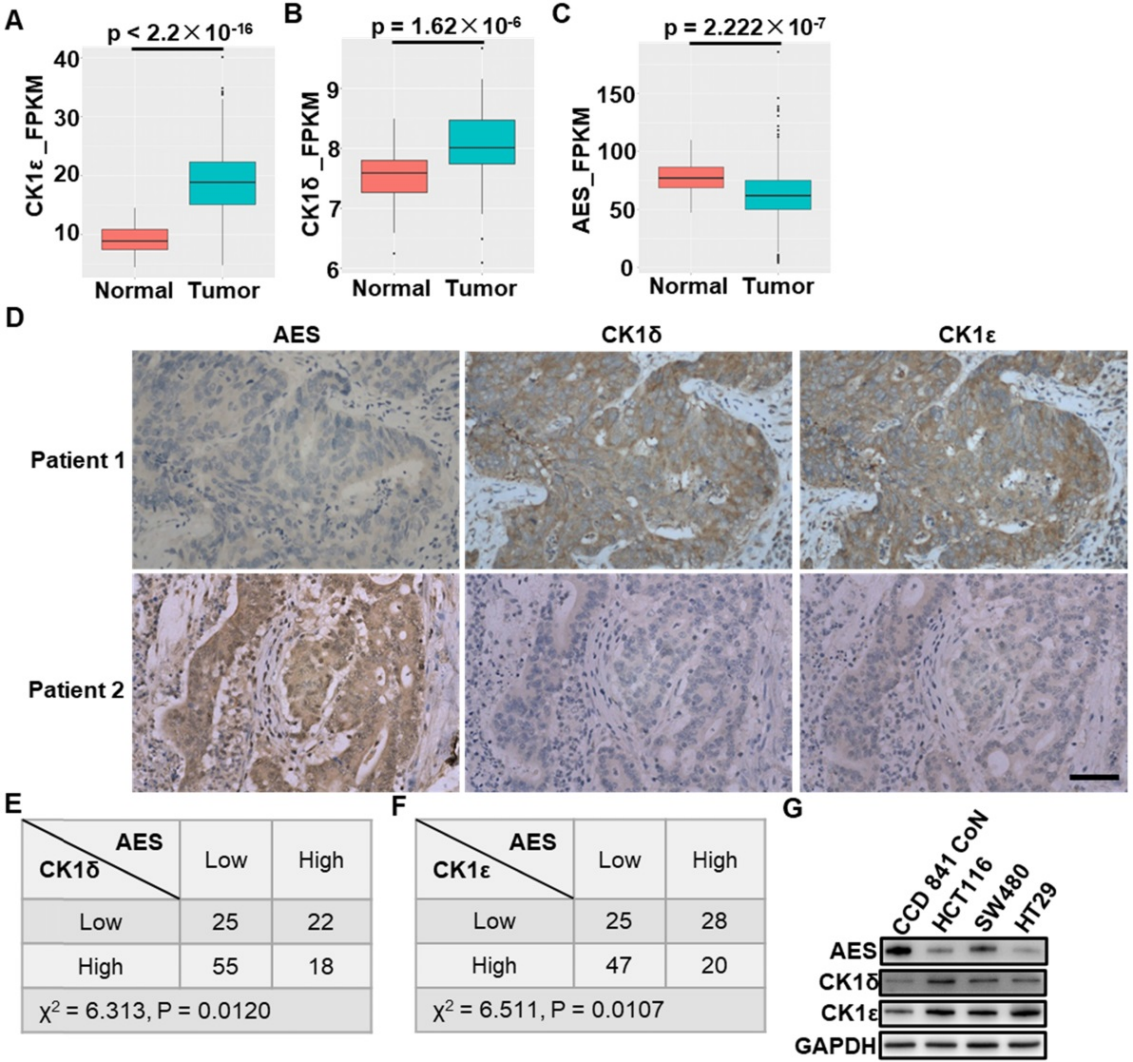
**The expression of CK1δ or CK1ε is inversely correlated with AES expression in human CRC specimens.** (**A-C**) The mRNA expression of CK1ε (**A**), CK1δ (**B**) and AES (**C**) in colon adenocarcinomas and non-malignant adjacent colon tissues using data from the Cancer Genome Atlas (TCGA) database. (**D**) Representative images of IHC staining to detect the expression of AES, CK1δ and CK1ε in CRC specimens. Scale bar, 50 μm. (**E**) The expression of AES was inversely related to CK1δ levels in CRC specimens (P < 0.05, χ2 tests). CK1δ and AES expression was detected by IHC staining in CRC specimens. The IHC staining for AES and CK1δ was scored as high or low based on their IHC expression levels. (**F**) Similar to (**E**) except that CK1ε expression was detected. The expression of AES was inversely related to CK1ε levels in CRC specimens (P < 0.05, χ2 tests). (**G**) The expression of AES, CK1δ and CK1ε in normal colon epithelial cell (CCD 841 CoN) and colon cancer cells (HCT116, SW480, HT29) were detected by WB.

We first evaluated the expression of AES, CK1δ and CK1ε in colon adenocarcinomas and non-malignant adjacent colon tissues using data from The Cancer Genome Atlas (TCGA) and GEO database (GSE20916) 41. These data showed that CK1ε and CK1δ expression was significantly up-regulated in colon cancers in comparison with non-malignant adjacent colon tissues, while lower expression of AES was found in colon cancer tissues (Figure 1A-C). Interestingly, in cancer specimens obtained from CRC patients, we observed a strong inverse relationship between the expression of AES and the expression of CK1δ or CK1ε. The decreased levels of AES were found to be associated with increased levels of CK1δ or CK1ε, while high levels of AES were accompanied by low levels of CK1δ or CK1ε (Figure 1D-F). Furthermore, the expression of AES, CK1δ and CK1ε in normal colon epithelial cells (CCD 841 CoN) and colon cancer cells (HCT116, SW480 and HT29) were detected. The colon cancer cells have been shown to have higher levels of CK1δ/ε and lower levels of AES than normal colon epithelial cells (Figure 1G). Concerning AES is known as a metastasis suppressor gene for CRC, we speculated that CK1δ and CK1ε may be relevant to the metastasis of CRC through regulating AES expression.

### CK1δ/ε physically interacts with AES and regulates its stability via the ubiquitin-proteasome pathway

**Figure 2:**
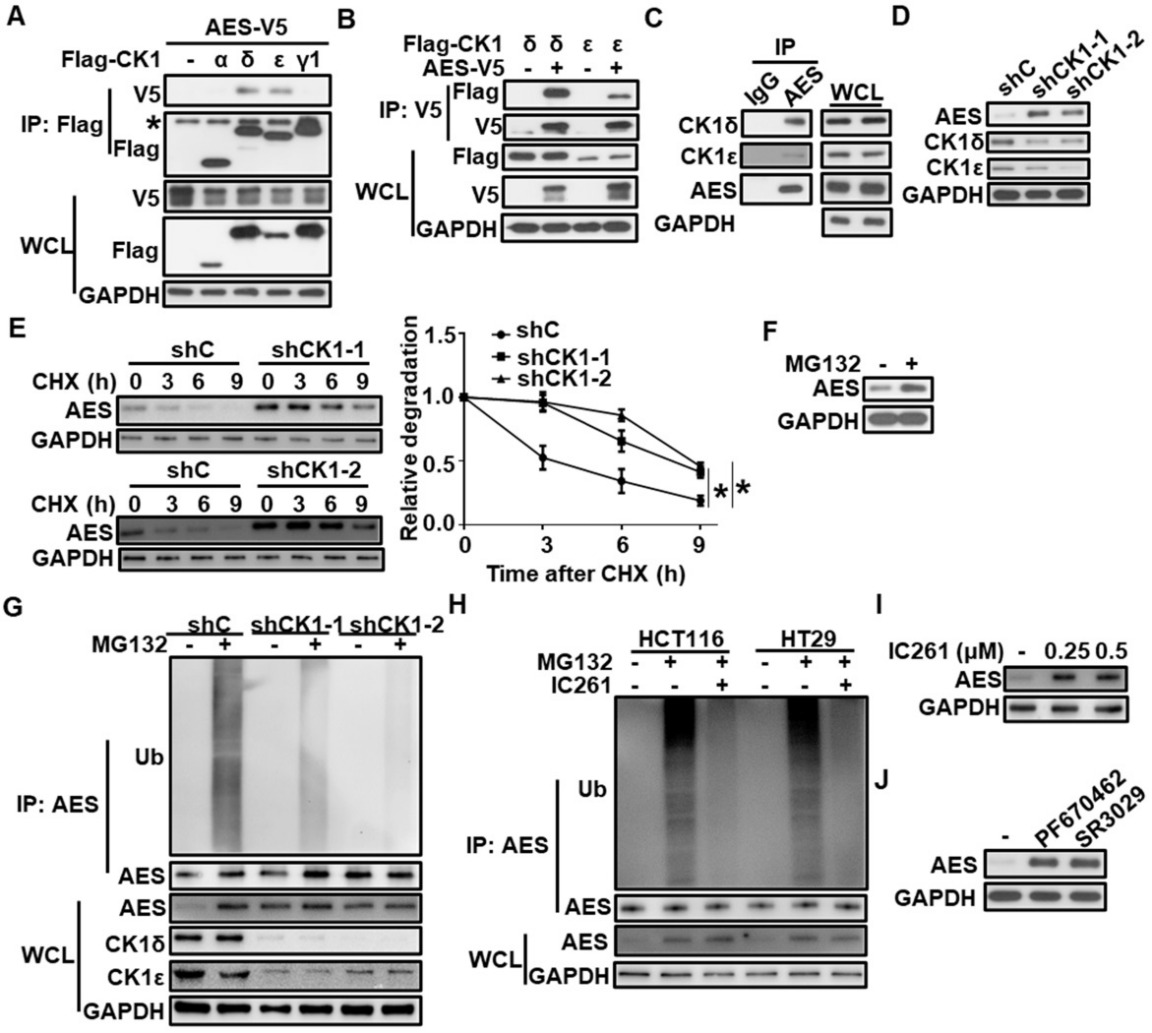
**CK1δ/ε interacts with AES and regulates AES stability.** (**A**) HEK293T cells were transfected with AES-V5 plasmid along with expression vectors for Flag-CK1α, Flag-CK1δ, Flag-CK1ε, and Flag-CK1γ1, respectively. Cell lysates were immunoprecipitated with anti-Flag M2 beads. The interaction of AES with different CK1 isoforms was detected by immunoblot analysis. The asterisk represents the IgG heavy chain. (**B**) HEK293T cells were transfected with AES-V5 plasmid together with expression vectors for Flag-CK1δ and Flag-CK1ε, respectively. Cell lysates were immunoprecipitated with anti-V5 agarose beads. The interaction of AES with CK1δ or CK1ε was visualized by immunoblotting. (**C**) Cell lysates from HCT116 cells were subjected to IP with IgG or anti-AES antibody. The interaction of AES with CK1δ or CK1ε was visualized by immunoblotting. (**D**) HCT116 cells were infected with shC (control), shCK1δ-1/shCK1ε-1 mixture (shCK1-1), or shCK1δ-2/shCK1ε-2 mixture (shCK1-2) lentivirus, then cell lysates were subjected to immunoblotting with the indicated antibodies. (**E**) HCT116 cells were infected with shC, shCK1δ-1/shCK1ε-1 mixture (shCK1-1), or shCK1δ-2/shCK1ε-2 mixture (shCK1-2) lentivirus and treated with 100 μg/ml CHX for the indicated periods of time. The expression of AES was detected by immunoblotting. The protein level of AES was quantitated by densitometry and normalized to GAPDH. (**F**) HCT116 cells were treated with 10 μM MG132 for 6 h, and the protein level of AES was detected by immunoblotting. (**G**) HCT116 cells were infected with shC, shCK1δ-1/shCK1ε-1 mixture (shCK1-1), or shCK1δ-2/shCK1ε-2 mixture (shCK1-2) lentivirus and treated with or without 10 μM MG132 for 6 h. Cell lysates were immunoprecipitated with AES antibody. The levels of AES-Ub and AES were detected by immunoblotting. (**H**) HCT116 or HT29 cells were treated with or without 0.25 μM IC261 for 24 h and 10 μM MG132 for 6 h before harvesting. Cell lysates were subjected to IP with AES antibody. The levels of AES-Ub and AES were detected by immunoblotting. (**I**) HCT116 cells were treated with DMSO or the indicated amounts of IC261 for 24 h. Cell lysates were subjected to immunoblotting with the indicated antibodies. (**J**) HCT116 cells were treated with DMSO, 2 μM PF670462 or 100 nM SR3029 for 24 h, then cell lysates were subjected to immunoblotting with the indicated antibodies. Values are shown as means ± SD (n = 3). *P < 0.05; Two-way ANOVA.

To test whether CK1 could physically interact with AES, HEK293T cells were transfected with AES-V5 expression vector along with expression plasmids for Flag-CK1α, Flag-CK1δ, Flag-CK1ε and Flag-CK1γ1, respectively. Cell extracts were collected for affinity purification by anti-Flag M2 agarose or anti-V5 agarose. As shown in Figure 2A-B, AES-V5 was coprecipitated with Flag-CK1δ or Flag-CK1ε, not with Flag-CK1α or Flag-CK1γ1, indicating that AES could specifically interact with CK1δ or CK1ε. Moreover, endogenous interaction between AES and CK1δ/ε was detected in HCT116 cells (Figure 2C). Although CK1δ/ε was clearly expressed in the cytoplasm and AES was predominantly localized in the nucleus, a low level of colocalization of CK1δ/ε and AES was observed in HCT116 cells (Figure S1A-B).

To check the effect of CK1δ/ε on AES expression, lentivirus-mediated shRNAs were used to silence the expression of CK1δ and CK1ε simultaneously in three CRC cell lines (HCT116, SW480 and HT29). Depletion of both CK1δ and CK1ε elevated the protein level of AES in three CRC cell lines without influencing AES mRNA level (Figure 2D and Figure S1C-E), suggesting that CK1δ/ε may mediate the AES stability. Treatment of HCT116 cells with the protein synthesis inhibitor cycloheximide (CHX) decreased the protein level of endogenous AES, and knockdown of CK1δ/ε extended the half-life of AES protein (Figure 2E). Moreover, treatment with the proteasome inhibitor MG132 enhanced the AES expression (Figure 2F). We also observed that polyubiquitinated AES (AES-Ub) was accumulated as high molecular weight smear bands in the presence of MG132, while knockdown of CK1δ/ε abolished the MG132-induced accumulation of AES-Ub (Figure 2G). In HCT116 and HT29 cells, the effect of MG132 was also blocked by the CK1 inhibitor IC261 (Figure 2H). Consistent with this, small molecular CK1 inhibitors (IC261, PF670462 and SR3029) clearly enhanced the protein level of AES in CRC cells (Figure 2I-J and Figure S1F-H). These results indicate that CK1δ/ε may mediate the degradation of AES via the ubiquitin-proteasome pathway.

### SKP2 interacts with AES and regulates the degradation of AES

**Figure 3:**
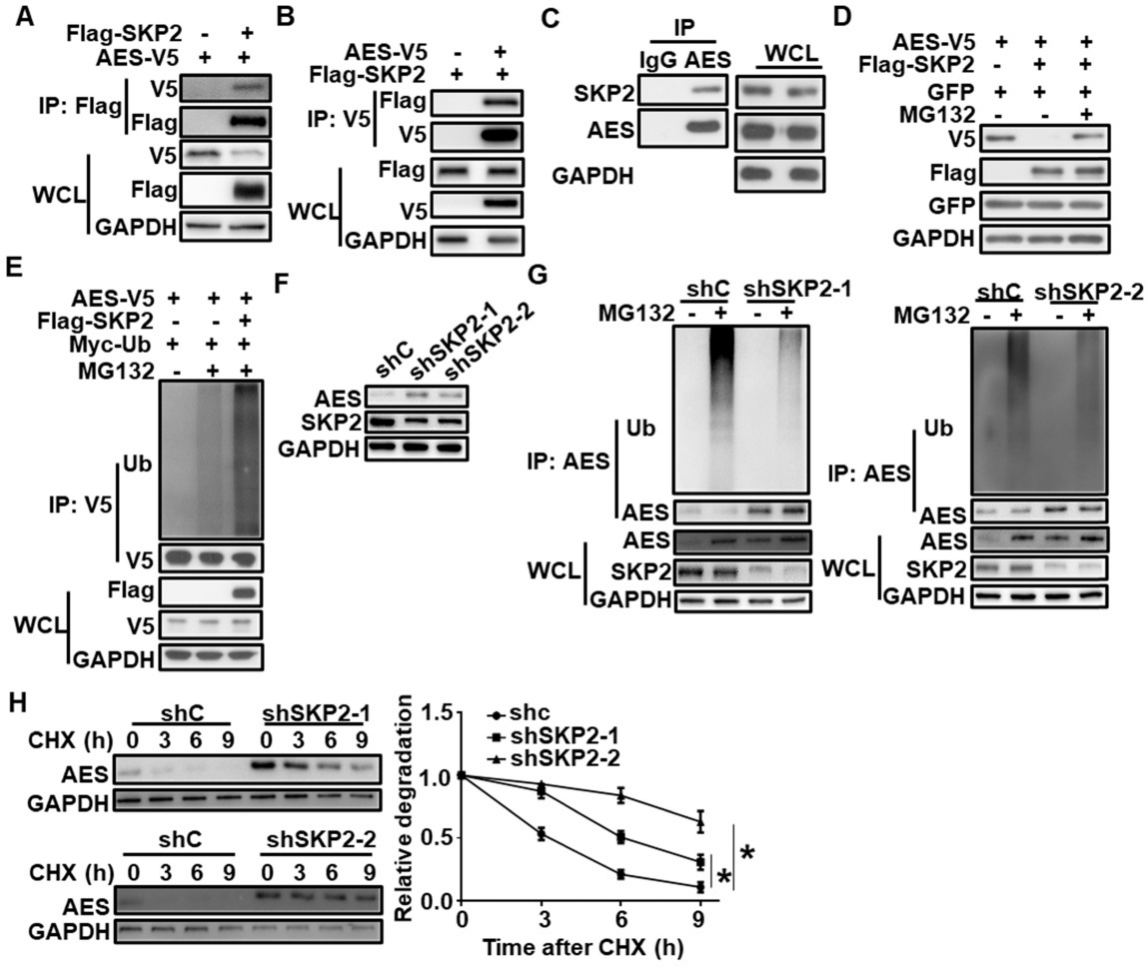
**SKP2 interacts with AES and mediates its degradation.** (**A**) HEK293T cells were transfected with AES-V5 plasmid along with expression vector for Flag-SKP2, then cells were lysed and subjected to IP with anti-Flag M2 beads. Immunoblot analysis was performed with the indicated antibodies. (**B**) Similar to (**A**) except anti-V5 agarose beads were used in Co-IP. (**C**) Cell lysates from HCT116 cells were subjected to IP with IgG or anti-AES antibody. Immunoblot analysis was performed with the indicated antibodies. (**D**) HEK293T cells were transfected with the indicated expression plasmids and treated with or without 10 μM MG132 for 6 h before harvesting. Cell lysates were subjected to immunoblotting with the indicated antibodies. The plasmid pEGFP-N1 was used to monitor transfection efficiency. (**E**) HEK293T cells were transfected with the indicated expression plasmids and treated with or without 10 μM MG132 for 6 h before harvesting. Cell lysates were immunoprecipitated with anti-V5 agarose beads. The levels of AES-Ub and AES were detected by immunoblotting. (**F**) HCT116 cells were infected with shC, shSKP2-1, or shSKP2-2 lentivirus, then cell lysates were subjected to immunoblotting with the indicated antibodies. (**G**) HCT116 cells were infected with shC, shSKP2-1 or shSKP2-2 lentivirus and treated with or without 10 μM MG132 for 6 h, then cell lysates were immunoprecipitated with AES antibody. The levels of AES-Ub and AES were detected by immunoblotting. (**H**) HCT116 cells were infected with shC, shSKP2-1, or shSKP2-2 lentivirus and treated with 100 μg/ml CHX for the indicated times. The expression of AES was detected by immunoblotting. The protein level of AES was quantitated by densitometry and normalized to GAPDH. Values are shown as means ± SD (n = 3). *P < 0.05; Two-way ANOVA.

Based on the UbiBrowser database 42, SKP2 was identified as a potential E3 ubiquitin ligase for AES. To test the interaction of SKP2 with AES, coimmunoprecipitation assay was conducted using HEK293T cells that were transiently transfected with Flag-tagged SKP2 and V5-tagged AES. The results showed that SKP2 could interact with AES (Figure 3A-B). Endogenous interaction between SKP2 and AES was also detected in HCT116 cells by coimmunoprecipitation analysis (Figure 3C).

We further showed that the overexpression of SKP2 in HEK293T cells reduced exogenous AES level, while the effects of SKP2 were rescued by MG132, suggesting that AES was degraded by the proteasome pathway (Figure 3D). Consistently, SKP2 coexpression significantly induced AES polyubiquitination (Figure 3E). Moreover, depletion of SKP2 in HCT116 cells caused an increase in AES protein level, and a decrease in the level of AES-Ub without influencing AES mRNA level (Figure 3F-G and Figure S2A). Additionally, SKP2 knockdown markedly increase the half-life of AES (Figure 3H). Notably, SKP2 expression was found to be significantly up-regulated in colon cancers in comparison with non-malignant adjacent colon tissues and its expression level was inversely correlated with AES expression, as demonstrated by bioinformatics analysis of TCGA database (Figure S2B-C). Giving that SKP2 is a known ubiquitin E3 ligase, SKP2 may catalyze the transfer ubiquitin from E2 to AES protein, resulting in the ubiquitination and degradation of AES.

### CK1δ/ε is required for SKP2/AES interaction and SKP2-mediated degradation of AES

**Figure 4:**
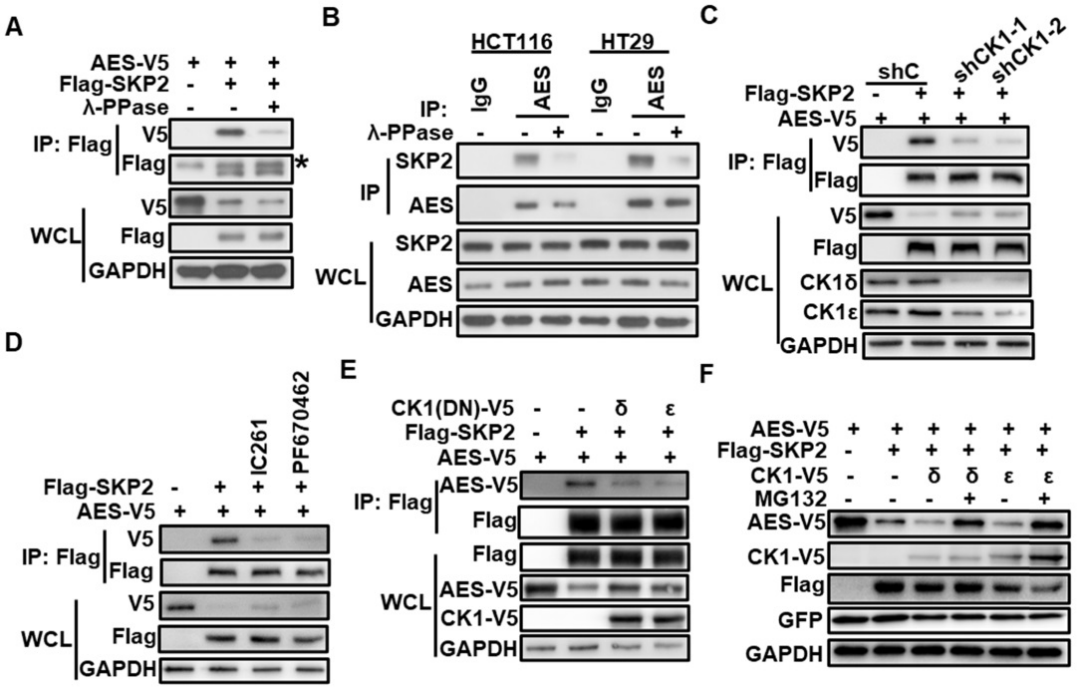
**CK1δ/ε is required for the interaction of SKP2 with AES and SKP2-mediated AES degradation.** (**A**) HEK293T cells were transfected with the indicated expression plasmids and cell lysates were treated with or without λ-PPase before immunoprecipitating with anti-Flag M2 beads. Immunoblot analysis was performed with the indicated antibodies. The asterisk represents the IgG heavy chain. (**B**) The lysates from HCT116 and HT29 cells were treated with or without λ-PPase before immunoprecipitating with anti-AES antibody. Immunoblot analysis was performed with the indicated antibodies. (**C**) HEK293T cells were infected with shC, shCK1δ/ε-1, or shCK1δ/ε-2 lentivirus, followed by transfection with the indicated plasmids. Cell lysates were immunoprecipitated with anti-Flag M2 beads. The interaction of AES with SKP2 was detected by immunoblotting. (**D**) HEK293T cells were transfected with the indicated plasmids followed by treatment with DMSO, 0.25 μM IC261, or 2 μM PF670462 for 24 h. Cell lysates were subjected to IP with anti-Flag M2 beads. Immunoblot analysis was performed to detect the interaction between AES and SKP2. (**E**) HEK293T cells were transfected with the indicated plasmids, and then cell lysates were subjected to IP with anti-Flag M2 beads. Immunoblot analysis was used to detect the interaction between AES and SKP2 in the absence or presence of dominant negative mutant of CK1δ or CK1ε. Anti-V5 antibody was used to detect AES-V5 and CK1-V5. (**F**) HEK293T cells were transfected with the indicated plasmids and treated with or without 10 μM MG132 for 6 h. Cells lysates were subjected to immunoblotting with the indicated antibodies. Anti-V5 antibody was used to detect AES-V5 and CK1-V5.

Interestingly, we noted that the interaction between SKP2 and AES was diminished upon treatment with λ-phosphatase (λ-PPase), suggesting that SKP2 might mediate AES stability in a phosphorylation-dependent manner (Figure 4A-B). We next examined the effect of CK1δ/ε on the interaction between SKP2 and AES in HEK293T cells. Depletion of CK1δ/ε abolished the interaction between SKP2 and AES (Figure 4C). CK1 inhibitors (IC261 and PF670462) also abrogated the association of SKP2 with AES (Figure 4D). Furthermore, the overexpression of kinase-dead dominant negative CK1δ/ε dramatically reduced the interaction between SKP2 and AES (Figure 4E). Consistently, the overexpression of CK1δ or CK1ε markedly enhanced the effect of SKP2 on AES expression, while MG132 treatment rescued AES expression (Figure 4F). These results indicate that CK1δ/ε is clearly involved in SKP2-mediated degradation of AES.

### Either CK1δ/ε or SKP2 interacts with AES at its Q domain, and CK1δ/ε phosphorylates AES at Ser121

**Figure 5:**
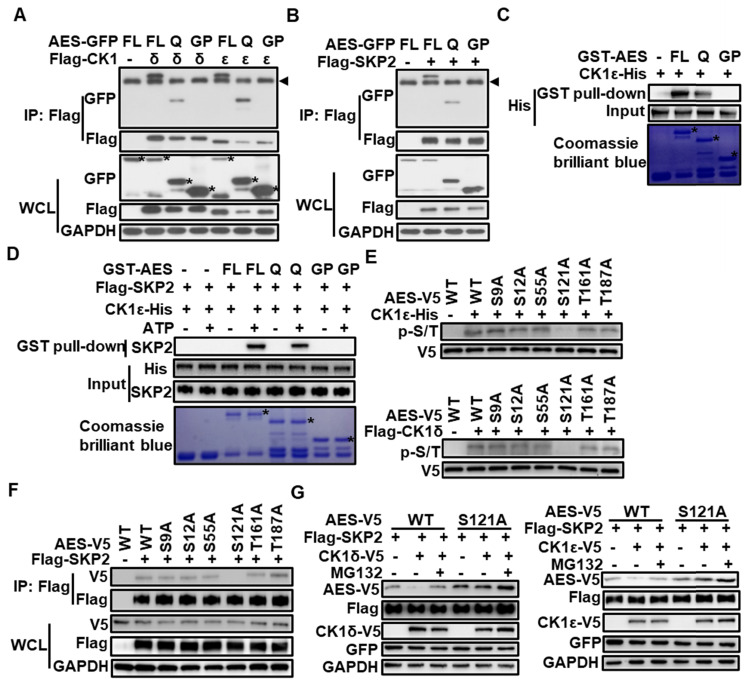
**CK1δ/ε phosphorylates AES at Ser121 to regulate the interaction of SKP2 with AES and the SKP2-mediated degradation of AES.** (**A**) HEK293T cells were transfected with expression plasmids encoding GFP tagged wild-type AES, and its C- or N-terminal truncation mutations (FL, full length; Q, Q domain; GP, GP domain) along with Flag-CK1δ or Flag-CK1ε vector, respectively. Cell lysates were immunoprecipitated with anti-Flag M2 beads. Immunoblot analysis was used to detect the interaction of CK1 with AES and its mutants. The arrow head represents the IgG heavy chain. The asterisks represent full length, Q domain or GP domain of AES. (**B**) Similar to (**A**) except that Flag-SKP2 plasmid, not Flag-CK1δ or Flag-CK1ε plasmids, was transfected into HEK293T cells. The arrow head represents the IgG heavy chain. (**C**) *In vitro* GST pulldown assay was performed using purified GST-AES fragments and CK1ε-His from *E. coli*. GST fusion proteins were shown by Coomassie brilliant blue staining and GST was used as a negative control. CK1ε-His was detected by immunoblotting using anti-His antibody. The asterisk represents the GST fusion proteins. (**D**) *In vitro* GST pulldown assay was performed. GST-AES fragments and CK1ε-His were purified from *E. coli.* Flag-SKP2 was purified from HEK293T cells transfected with expression plasmid encoding Flag-SKP2. ATP (1 mM) was added as indicated. GST proteins were shown by Coomassie brilliant blue staining and GST was used as a negative control. CK1ε-His and Flag-SKP2 was detected by immunoblotting using anti-His-Tag and anti-SKP2 antibody, respectively. The asterisk represents the GST fusion proteins. (**E**) V5-tagged wild-type AES (WT) and its point mutants were immunoprecipitated from transfected HEK293T cells with anti-V5 agarose beads. After λ-PPase treatment, immunoprecipitated proteins were subjected to reaction with CK1ε-His purified from *E. coli* or Flag-CK1δ purified from HEK293T cells transfected with Flag-CK1δ expression vector in the presence of 1 mM ATP. The phosphorylated AES was detected by immunoblotting using anti-phospho-Ser/Thr (p-S/T) antibody. (**F**) HEK293T cells were transfected with expression vectors for V5-tagged wild-type AES (WT) and its point mutants along with empty vector or Flag-SKP2 plasmid. Cell lysates were subjected to immunoprecipitation with anti-Flag M2 beads. Immunoblot analysis was used to detect the interaction of Flag-SKP2 with AES or its point mutants. (**G**) HEK293T cells were transfected with the expression plasmids for AES or its S121A mutant together with Flag-SKP2 and CK1ε-V5 or CK1δ-V5 plasmids. The plasmid pEGFP-N1 was used to monitor transfection efficiency. Cell lysates were subjected to immunoblotting with the indicated antibodies. As indicated, cells were treated with 10 μM MG132 for 6 h before harvesting.

The AES protein consists of an N-terminal glutamine-rich (Q) domain and a C-terminal glycine/ proline-rich (GP) domain. The highly conserved Q domain is able to mediate multimerization between AES and other TLE/GRG family members as well as interaction with TCF/LEF transcription factors 43. To determine the domain responsible for CK1δ/ε or SKP2 interaction, C- and N-terminal truncation mutants of AES were constructed and fused to the C-terminus of GFP or GST tag, respectively. Coimmunoprecipitation analysis showed that either CK1δ/ε or SKP2 could interact with full-length AES and AES-Q domain, but not AES-GP domain (Figure 5A-B). Glutathione S-transferase (GST) pull-down assays revealed that the AES-Q domain was the primary binding domain for CK1ε or CK1δ (Figure 5C and Figure S3). Figure 5D showed that SKP2 could interact with either full-length AES or AES-Q domain but not AES-GP domain in a phosphorylation-dependent fashion. Furthermore, treatment with λ-PPase abolished the association of SKP2 with AES-Q domain, suggesting that phosphorylation may modulate the interaction between SKP2 and AES-Q domain (Figure S2D).

CK1 phosphorylates its substrates that have a consensus sequence of D/EXXS or S/T-PO_4_XXS/T 44. Sequence analysis of human AES revealed six potential CK1 phosphorylation sites. To identify the exact site phosphorylated by CK1δ/ε in AES, we generated S9A, S12A, S55A, S121A, T161A and T187A AES mutants in constructs expressing V5-tagged AES. Wild-type and mutated AES were transiently transfected into HEK293T cells and immunoprecipitated by anti-V5 agarose beads for *in vitro* kinase assay. Purified CK1δ/ε-induced AES phosphorylation was completely blocked when Ser121 was mutated to alanine (Figure 5E), indicating that Ser121 is a critical CK1 phosphorylated site. Notably, AES mutant (S121A) failed to interact with SKP2 in coimmunoprecipitation assay (Figure 5F). Ectopic expression of SKP2 and CK1ε or CK1δ reduced the expression of wild-type AES, but had no effect on the expression of AES mutant (S121A) (Figure 5G). These findings indicate that CK1δ/ε may phosphorylate AES at Ser121, which required for SKP2-mediated degradation of AES.

### CK1ε inhibits AES-mediated effect on Wnt and Notch signaling

**Figure 6:**
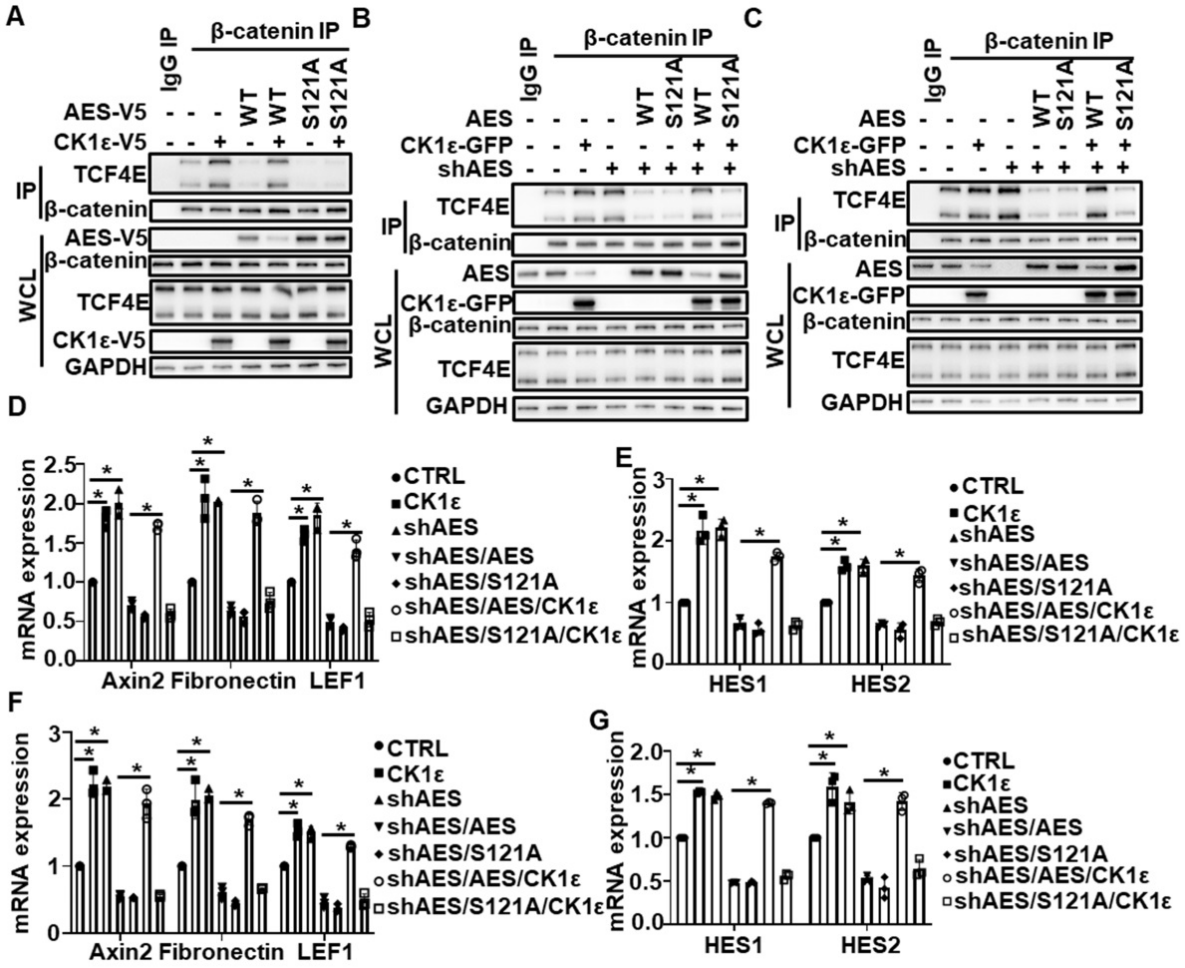
**CK1ε antagonizes AES-mediated effect on Wnt and Notch signaling.** (**A**) HEK293T cells were transfected with the indicated plasmids. Cell lysates were immunoprecipitated with IgG or anti-β-catenin agarose beads. The interaction of β-catenin with TCF4E was detected by immunoblotting. (**B** and** C**) HCT116 (**B**) and HT29 (**C**) cells were infected with the indicated lentivirus, respectively. Cell lysates were subjected to IP with IgG or anti-β-catenin agarose beads. Immunoblot analysis was performed to detect the interaction between β-catenin and TCF4E. (**D-G**) HCT116 (**D** and** E**) and HT29 (**F** and** G**) cells were infected with the indicated lentivirus. Then total RNA was extracted and real-time PCR was performed to detect the mRNA expression. Quantification of mRNA level was normalized to GAPDH. Values are shown as means ± SD (n = 3). *P < 0.05; Student's t test.

We noted that both CK1ε and CK1δ expression was significantly up-regulated in colon cancers in comparison with non-malignant adjacent colon tissues, but the difference was much more significant for CK1ε (Figure 1A-B). Concerning that CK1ε shares much functional redundancy with CK1δ due to a high homologous sequence, CK1ε was selected for follow-up experiments. Previous studies have shown that AES could inhibit Wnt and Notch signaling and modulate gene expression by interacting with transcriptional factors, such as TCF/LEF family members 11, 12. We next assess the effect of CK1*ε* on AES-mediated inhibition of Wnt and Notch signaling. As expected, ectopic expression of either wild-type AES or its point mutant (S121A) abolished the interaction of TCF4E with β-catenin, and CK1ε expression reversed the effect of wild-type AES on this interaction without affecting the mutant-mediated inhibition (Figure 6A). Consistently, the interaction between TCF4E and β-catenin was enhanced in AES knockdown CRC cells (HCT116 and HT29), while this interaction was rescued by reintroduction and expression of either wild-type or mutant (S121A) AES (Figure 6B-C). Ectopic expression of CK1ε restored the effect of wild-type AES but not its mutant (S121A) (Figure 6B-C). Importantly, increased mRNA expression of Wnt target genes (Axin2, Fibronectin and LEF1) and Notch target genes (HES1 and HES2) was detected by real-time PCR in CRC cells with AES knockdown, while reintroduction of wild-type or mutant (S121A) AES reversed the expression of Wnt target genes and Notch target genes. Simultaneous overexpression of CK1ε rescued the effect of wild-type but not mutant (S121A) AES (Figure 6D-G). These results suggest that CK1 antagonizes the inhibitory effect of AES on Wnt and Notch probably through phosphorylating AES at Ser121 site.

### CK1ε abrogates the effects of AES on anchorage-independent growth, migration, invasion and sphere formation in CRC cells

**Figure 7:**
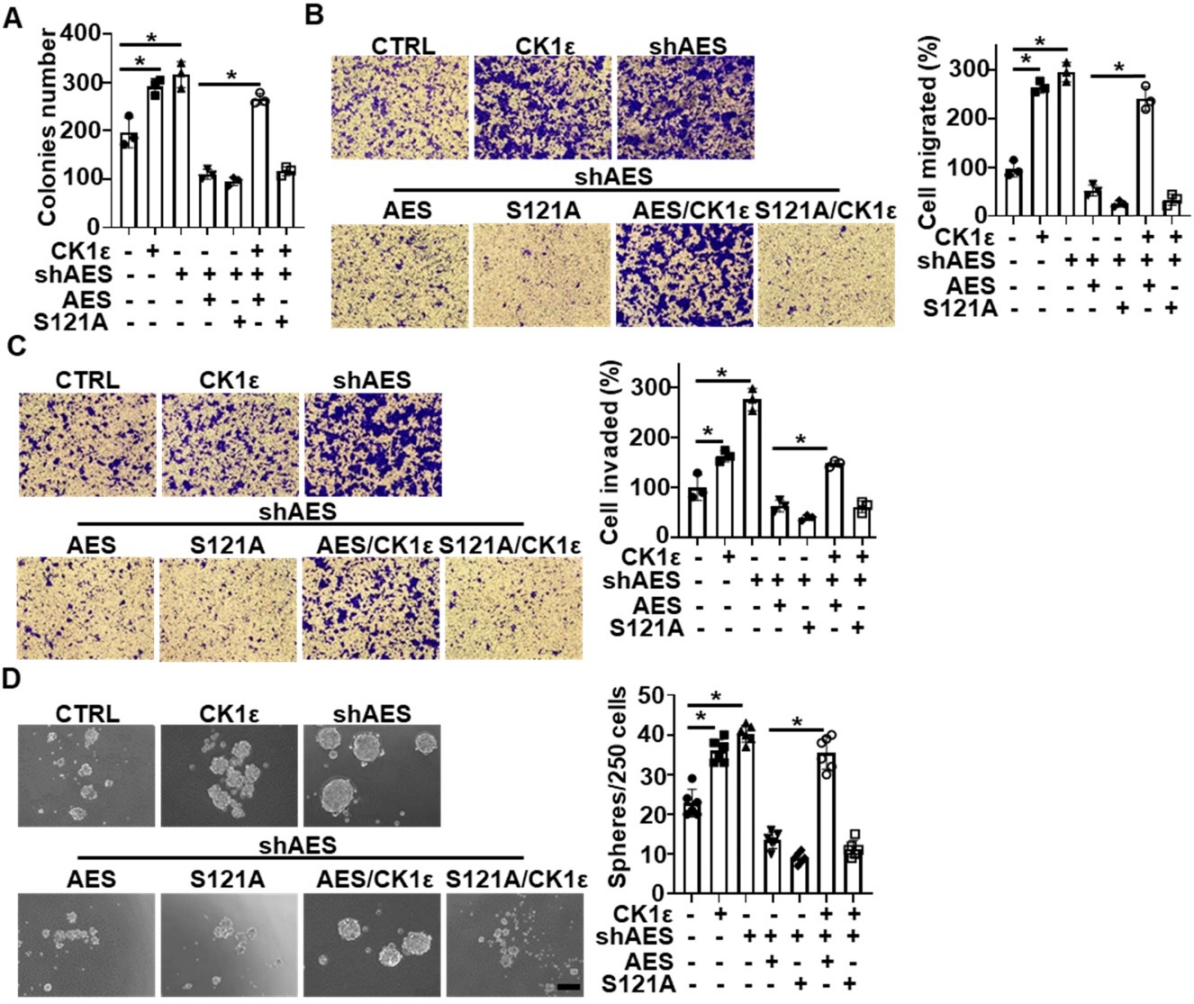
**CK1ε antagonizes the effect of AES on anchorage-independent growth, migration, invasion, and sphere formation in CRC cells.** HT29 cells were infected with the indicated lentivirus. (**A**) Soft agar colony formation assay was performed to evaluate the anchorage-independent growth. Graphical representation of quantitative data showed the relative number of colonies formed (n = 3). (**B** and** C**) Transwell assay was performed to evaluate cell migration (**B**) and invasion (**C**). Cells that migrated or invaded cells through transwells were stained and photomicrographed. Right panel: graphical representation of quantitative data showed the relative number of migrated (**B**) or invaded (**C**) cells (n = 3). (**D**) The sphere-forming ability was assessed in HT29 cells. Representative images of sphere formation were presented. Right panel: graphical representation of quantitative data showed the relative number of spheres formed (n = 6). Scale bar, 200 μm. Values are shown as means ± SD. *P < 0.05; Student's t test.

As previously reported, high expression of CK1 is correlated with poor prognosis in CRC patients, and AES functions as a metastasis repressor in CRC 11, 45, 46. We next investigated the effects of AES and CK1 expression on the biological behaviors of CRC cells (HT29 and HCT116). Depletion of AES enhanced the anchorage-independent growth, migration, invasion and sphere formation of CRC cells, while reintroduction of wild-type or mutant (S121A) AES reversed the effect of AES depletion. Simultaneous overexpression of CK1ε reversed the effects of wild-type AES but not mutant (S121A) (Figure 7A-D and Figure S4A-D). Together, these findings suggest that CK1 is involved in AES-mediated anchorage-independent growth, migration, invasion and sphere-forming ability of CRC cells.

In order to determine the effects of some Wnt target genes (LEF1, CD44 and LGR5) on AES-mediated biological behaviors in CRC cells, the expression of LEF1, CD44 and LGR5 was simultaneously knocked down in HCT116 and HT29 cells. As shown in Figure S5, *knockdown* of LEF1, CD44 and LGR5 abrogated the effects of AES knockdown on anchorage-independent growth, migration, invasion and sphere formation in CRC cells, respectively (Figure S5). The results indicate that AES may exert its biological effects on CRC cells via regulating the Wnt signaling pathway.

### CK1ε antagonizes the suppressive effect of AES on APC^min/+^ colorectal tumor organoid growth and CRC metastasis

**Figure 8:**
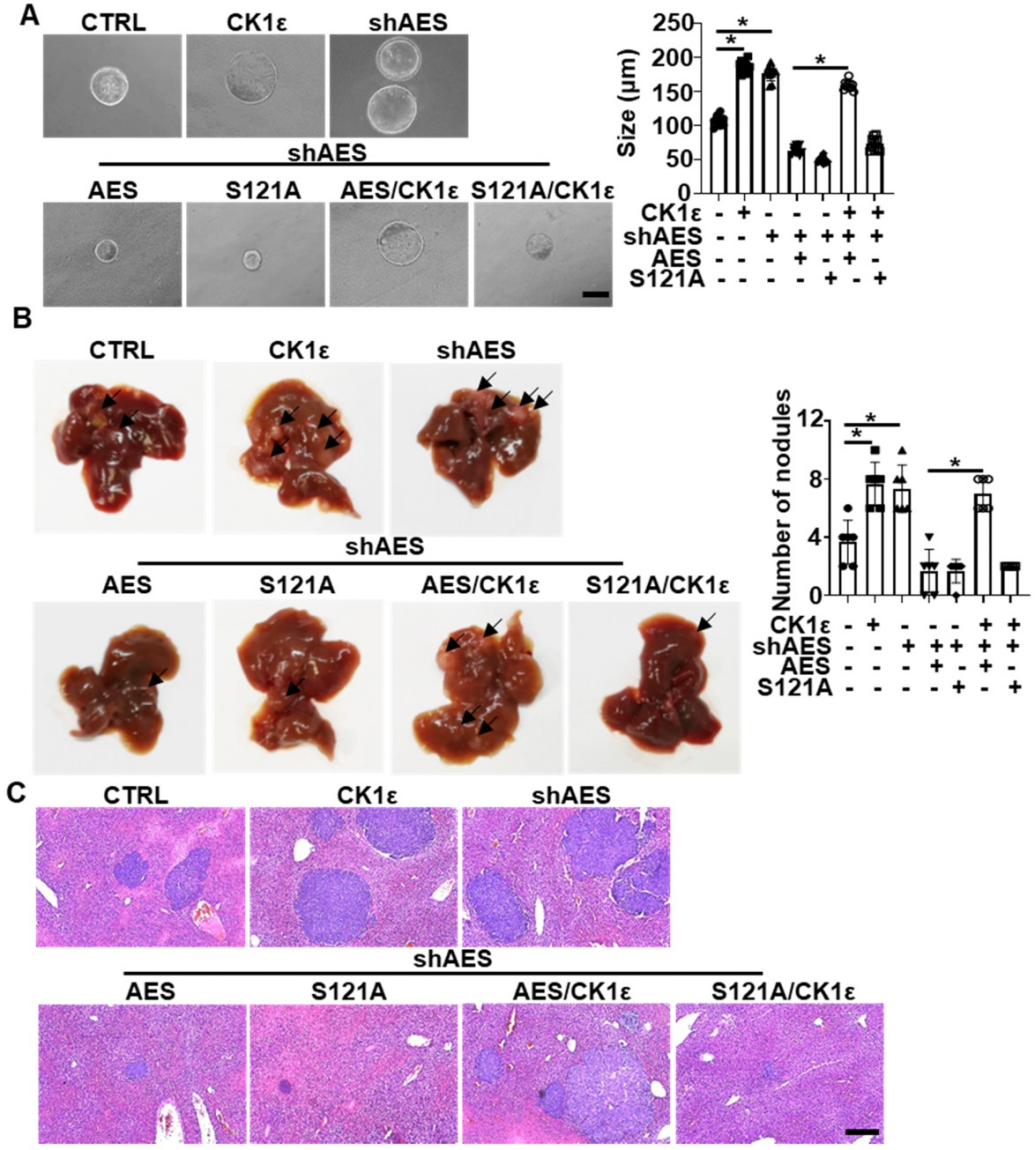
**CK1ε antagonizes the effect of AES on APC^min/+^ colorectal tumor organoid growth and CRC metastasis *in vivo*.** (**A**) Morphology of colorectal tumor organoids from B6-APC^min/+^ mice infected with the indicated lentivirus. Right panel: graphical representation of quantitative data showed the relative size of organoid (n = 10). Scale bar, 200 μm. (**B** and** C**) HCT116 cells were infected with the indicated lentivirus and intrasplenically injected into nude mice, respectively. (**B**) Representative images of liver metastasis (n = 6). Right panel: graphical representation of quantitative data showed the relative rate of liver metastasis. (**C**) H&E staining. Scale bar, 200 μm. Values are shown as means ± SD. *P < 0.05; Student's t test.

To explore the effects of CK1 on AES-mediated tumor growth, colorectal tumor organoids from APC^min/+^ transgenic mice were infected with the indicated lentivirus. Knockdown of AES promoted organoid growth, whereas reintroduction of either wild-type or mutant (S121A) AES reversed AES knockdown-mediated effects on organoid growth. Simultaneously ectopic expression of CK1ε rescued the effect of wild-type AES but not mutant (S121A) (Figure 8A). Importantly, AES knockdown in HCT116 cells enhanced liver metastasis in nude mice, whereas reintroduction of either wild-type or mutant (S121A) AES reversed the effect of AES knockdown on liver metastasis. Overexpression of CK1ε promoted liver metastasis in HCT116 cells overexpressing wild-type AES but not mutant (S121A) (Figure 8B-C). These results suggest that CK1 antagonizes AES-mediated suppression of tumorigenic and metastatic potentials possibly through phosphorylation of AES at Ser121.

**Figure 9:**
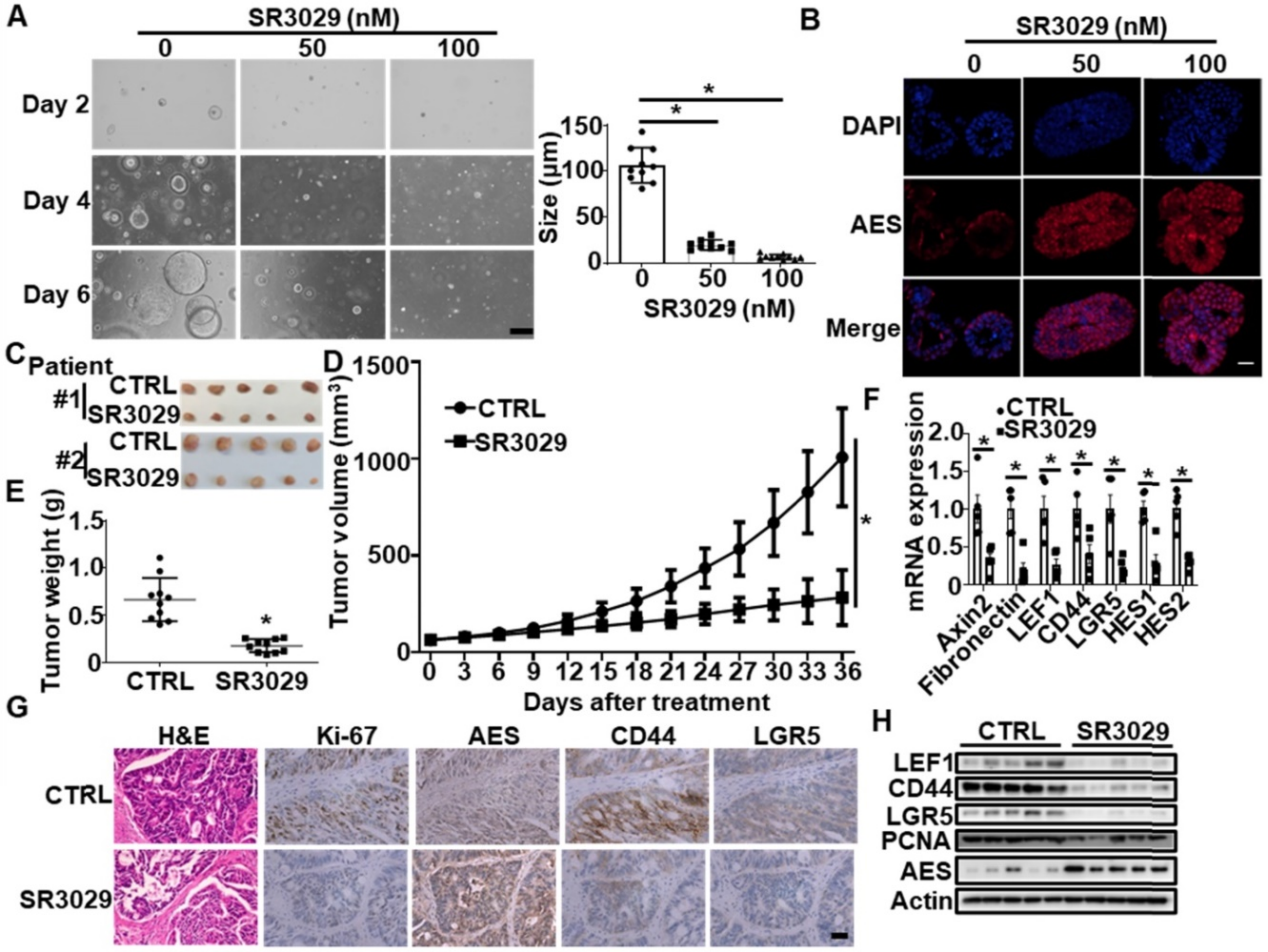
**SR3029 represses the growth of APC^min/+^ colorectal tumor organoids and patient-derived colorectal tumor xenografts (PDTX) through enhancing AES expression.** (**A**) Morphology of colorectal tumor organoids from B6-APC^min/+^ mice treated with SR3029. The organoids were treated with the indicated amounts of SR3029 for 24 h. Then SR3029 was removed and the organoids were grown in normal medium for another 5 days before photomicrographed. Right panel: graphical representation of quantitative data showed the relative size of organoid. Scale bar, 200 μm. (**B**) AES staining of colorectal tumor organoids treated with or without SR3029. Scale bar, 50 μm. (**C**) Representative images of tumors from the control and SR3029-treated PDTX. (**D**) Mean tumor volume (n = 10). (**E**) Mean tumor weight (n = 10). (**F**) Total RNA was extracted from tumor samples treated with or without SR3029 and real-time PCR was performed to detect the mRNA expression of Wnt target genes (Axin2, Fibronectin and LEF1), Wnt-related stemness marker genes (CD44 and LGR5) and Notch target genes (HES1 and HES2). Quantification of mRNA level was normalized to GAPDH (n = 5). (**G**) H&E, Ki-67, AES, CD44, and LGR5 staining of control and SR3029-treated PDTX. Scale bar, 50 μm. (**H**) The expression levels of LEF1, CD44, LGR5, PCNA and AES in tumor samples from the control and SR3029-treated PDTX were detected by immunoblotting. Values are shown as means ± SD. *P < 0.05; Two-way ANOVA for (**D**); Student's t test for (**A**), (**E**) and (**F**).

In the PDTX model, administration of SR3029 obviously delayed tumor growth (Figure 9C-E), concomitant with an increased protein level of AES and a decreased expression of Wnt target genes (Axin2, Fibronectin and LEF1), stemness marker genes (CD44 and LGR5) and Notch target genes (HES1 and HES2) (Figure 9F-H). Collectively, these findings suggest that SR3029 may inhibit CRC growth through stabilizing AES.

## Discussion

Metastatic disease is responsible for the majority of cancer deaths. In CRC, the most common site of metastatic spread is the liver 2, 3. Metastasis is a complex process that involves a variety of different genes and signaling pathways 47. AES has been found to suppress local invasion and intravasation through inhibition of the Notch pathway, leading to preventing the metastatic spread of endogenous tumors. AES could inhibit the Notch pathway through blocking the Notch signaling transcription complex composed of the NOTCH intracellular domain (NICD), the transcription factor RBPJ, and the cofactor MAML 11. AES also inhibited HIF1α-mediated transcription as well as AR and BMP signaling 13, 14, 48. Furthermore, AES has been shown to inhibit the Wnt/β-catenin signaling cascade through interacting with TCF4 and disrupting β-catenin and TCF4 binding 12. Importantly, AES expression has been found to be downregulated in liver metastasis of CRC patients 11, 49. However, it remains unclear how the expression of AES is repressed in the invasive cancer cells. In the present study, we demonstrated that the stability of AES could be regulated by CK1δ/ε-AES axis in CRC cells. CK1δ/ε is able to interact with AES and phosphorylate AES at Ser121, accelerating SKP2-mediated ubiquitination and degradation of AES. Our results revealed a novel molecular mechanism involved in the regulation of AES expression in CRC cells.

CK1δ/ε has been reported to regulate the stability of multiple proteins, such as YAP and PER2 50, 51. The transcription coactivator YAP could be phosphorylated by Lats on Ser 381, and this phosphorylation provides the priming signal for CK1δ/ɛ to phosphorylate a phosphodegron, The phosphorylated phosphodegron recruits the SCF^β-TRCP^ E3 ubiquitin ligase, leading to the ubiquitination and degradation of YAP 50. Philpott et al. showed that PER2 stability was regulated by a two-state conformational switch in the CK1 activation loop which controls conformation of the kinase activation loop and determines which sites on mammalian PER2 are preferentially phosphorylated 51. It will be interesting to address whether SKP2 is involved in PER2 ubiquitination and degradation. Notably, PER2 has been shown to have tumor suppressive role through regulating the expression of multiple glycolytic genes, indicating that CK1δ/ε may be implicated in tumor-promoting glycolysis via the regulation of PER2 stability 52.

The ubiquitin-proteasome system (UPS) is a major mechanism of intracellular protein degradation and regulates diverse cellular functions. This system is composed of ubiquitin, ubiquitin-activating enzymes (E1), ubiquitin-conjugating enzymes (E2), ubiquitin ligases (E3), deubiquitinases, and proteasomes. The SCF is a well-characterized E3 ligase complex, containing S-phase kinase-associated protein 1 (SKP1), Ring box protein-1 (Rbx1), cullin 1 (Cul1), and multiple F-box proteins for substrate-recognizing including SKP2, Fbw7 and βTrCP. The SCF is responsible for catalyzing the ubiquitination of substrates for subsequent degradation in the 26S proteasome 53, 54. SKP2 has been shown to regulate the stability of co-activator-associated arginine methyltransferase 1 (CARM1), a crucial component of autophagy in mammals. Moreover, SKP2 expression was transcriptionally repressed by AMP-activated protein kinase (AMPK)-dependent phosphorylation of FOXO3a 55. Katona et al reported that inhibition of menin synergized with small molecule inhibitors of EGFR to suppress CRC via EGFR-independent and calcium-mediated repression of SKP2 transcription 56. In the present study, we found that SKP2 could interact with AES and induce the ubiquitination and degradation of AES. The SKP2-mediated degradation of AES was CK1δ/ε-dependent. CK1δ/ε may phosphorylate AES at Ser121 and phosphorylated AES were recognized by the F box protein SKP2, resulting in AES ubiquitination and proteasomal degradation. Interestingly, we noted that AES could interact with another SCF component Rbx1 (Figure S2E), suggesting that the SCF complex may participate the regulation of AES degradation.

Cancer stem cells (CSCs) are a small group of cancer cells which possess the capability to self-renew and differentiate into diverse cell types, and play a critical role in CRC recurrence, metastasis and therapeutic resistance 57, 58. Multiple CSC molecular markers have been identified in CRC, including LGR5 and CD44 59. Increasing evidence has demonstrated the activation of Wnt/β-catenin and Notch signaling has been involved in the growth and maintenance of colorectal CSCs 60. CK1δ/ε has been found to be required for Wnt-mediated intestinal stem cell maintenance 30. Li et al reported that the overexpression of SKP2 was associated with colorectal carcinogenesis and late metastasis to lymph nodes 61. Our results showed that the CK1δ/ε enhanced AES degradation via SKP2-mediated ubiquitin-proteasome pathway, resulting in the activation of Wnt and Notch signaling. By downregulating the expression of AES, CK1δ/ε promoted anchorage-independent growth, migration, invasion and sphere formation in CRC cells. CK1δ/ε also accelerated tumor growth and liver metastasis in CRC mouse models. In rescue experiments, knockdown of LEF1, LGR5 or CD44 reversed the increasing effect of anchorage-independent growth, migration, invasion and sphere formation in CRC cells due to AES depletion (Figure S5). Furthermore, a selective CK1ε/δ inhibitor SR3029 attenuated tumor growth through upregulating AES expression in APC^min/+^ colorectal tumor organoids and PDTX models. Notably, the expression of stemness marker genes (CD44 and LGR5) were downregulated by SR3029 in organoids and PDTX models. Taken together, these results indicated that the CK1δ/ε-AES axis may be critical for CRC metastasis and maintenance of stem cell functions, and blockade of this axis should have the potential for the treatment of CRC. In conclusion, the present study identified a CK1δ/ε-AES axis for the regulation of AES expression, and this axis is involved in the tumorigenesis and metastasis of CRC (Figure S7). Blocking this axis may provide a new opportunity for therapeutic intervention of CRC.

## Supplementary Material

Supplementary figures and tables.
