# Supplementary material for: The CK1δ/ε-AES axis regulates tumorigenesis and metastasis in colorectal cancer
Source: Theranostics. 2021 Mar 4;11(9):4421–35. doi: 10.7150/thno.53901 (PMC7977458; doi:10.7150/thno.53901)
Supplement: Supplementary file 1 — Supplementary figures and tables. [file thnov11p4421s1.pdf]

Supplementary Materials for

The CK1δ/ε-AES axis regulates tumorigenesis and metastasis in colorectal cancer

Zhongyuan Wang, Liang Zhou, Yejun Wang, Quanzhou Peng, Huan Li, Xin Zhang, Zijie Su, Jiaxing Song, Qi Sun, Sapna Sayed, Shanshan Liu, and Desheng Lu

The PDF file includes:

• Materials and Methods

• Fig. S1. CK1δ/ε regulates AES stability in CRC cells.

• Fig. S2. SKP2 interacts with AES Q domain and promotes the degradation of AES.

• Fig. S3. CK1δ phosphorylates AES to regulate the interaction of SKP2 with AES Q domain.

• Fig. S4. CK1ε antagonizes the effect of AES on anchorage-independent growth, migration, invasion, and sphere formation in CRC cells.

• Fig. S5. Some Wnt target genes antagonizes the effect of AES on anchorage-independent growth, migration, invasion, and sphere formation in CRC cells.

• Fig. S6. SR3029 has little effect on cell viability of $APC^{min/+}$ colorectal tumor organoids.

• Fig. S7. A proposed model for the regulation of AES stability by CK1δ/ε-SKP2 axis.

• Table S1. Primer sequences used for real-time PCR amplification.

• Table S2. Antibodies used in this study.

• Table S3. shRNA sequences used in this study.

Material and Methods

Cell culture

Human normal colon epithelial cell CCD 841 CoN, human colon cancer cell lines (HCT116, SW480 and HT29) and HEK293T cells were obtained from American Type Culture Collection (ATCC). All cells were maintained in DMEM supplemented with 10% fetal bovine serum (FBS) and 1% penicillin-streptomycin at 37 °C and 5% CO_2. All cells were tested for mycoplasma using a MycoFluor Mycoplasma Detection Kit (Thermo Fisher Scientific) and DNA fingerprinted with a PowerPlex 1.2 kit (Promega).

Plasmids

All the expression plasmids were constructed using the Flag-CMV2, pcDNA3.1-V5/His or EGFP-N1 vector. The shRNAs targeting human CK1δ and human CK1ε were inserted into pSilencer2.1-U6 hygro vector and then shuttled into FG12 vector. The shRNAs targeting human SKP2, human AES, mouse CK1δ, mouse CK1ε and mouse AES were cloned into pLKO.1-puro vector. The sequences of these shRNAs are shown in Supplementary Table S3. All the cloned sequences were confirmed by sequencing analysis.

Anchorage-independent growth assays

Each well of 12-well plates was pre-coated with 700 μl DMEM supplemented with 10% FBS and 0.7% agar. The cells were trypsinized and suspended in 0.35% agar in a complete DMEM at a density of 5,000 cells/ml, and then 300 μl of cell suspension were seeded into each well of the pre-coated plates in triplicate. The cells were then

maintained at 37 °C and 5% CO_2 for 2 weeks. Colonies were stained with 0.1% crystal violet before photographed. Colonies with diameter over than 0.1 mm were scored as positive.

Sphere formation assays

HCT116 cells were seeded at 250 cells/well in DMEM/F12 medium supplemented with 2% B-27, 10 ng/ml EGF, 10 ng/ml FGF and 10 μg/ml insulin in triplicate in an ultra-low attachment 24-well plate. After 10 days of growth, spheres with diameter over 50 μm were scored as positive and representative fields were photographed.

Cell migration and invasion assays

For migration assay, 2×10^5 cells in serum-free medium were cultured in upper chambers with 8-μm pore membrane, and the lower chambers of 24-well plates contained medium supplemented with 20% FBS. For invasion assay, the upper chambers with 8-μm pore membrane were coated with Matrigel before seeding cells. After incubation at 37°C for 24 hours, the unmigrated or uninvaded cells were removed and the migrated or invaded cells were stained with crystal violet and photomicrographed. Then the crystal violet was eluted by a 33% acetic acid solution and quantified by measuring the absorbance at 570 nm.

Cell viability assays

$APC^{min/+}$ colorectal tumor organoids were plated in Matrigel onto 96-well plates and

treated with DMSO or SR3029 at the indicated concentrations for 24 h. Then the cell viability assay was performed using the CellTiter-Glo 3D Cell Viability Assay Kit (Promega).

Supplementary Figure S1, Wang et al.

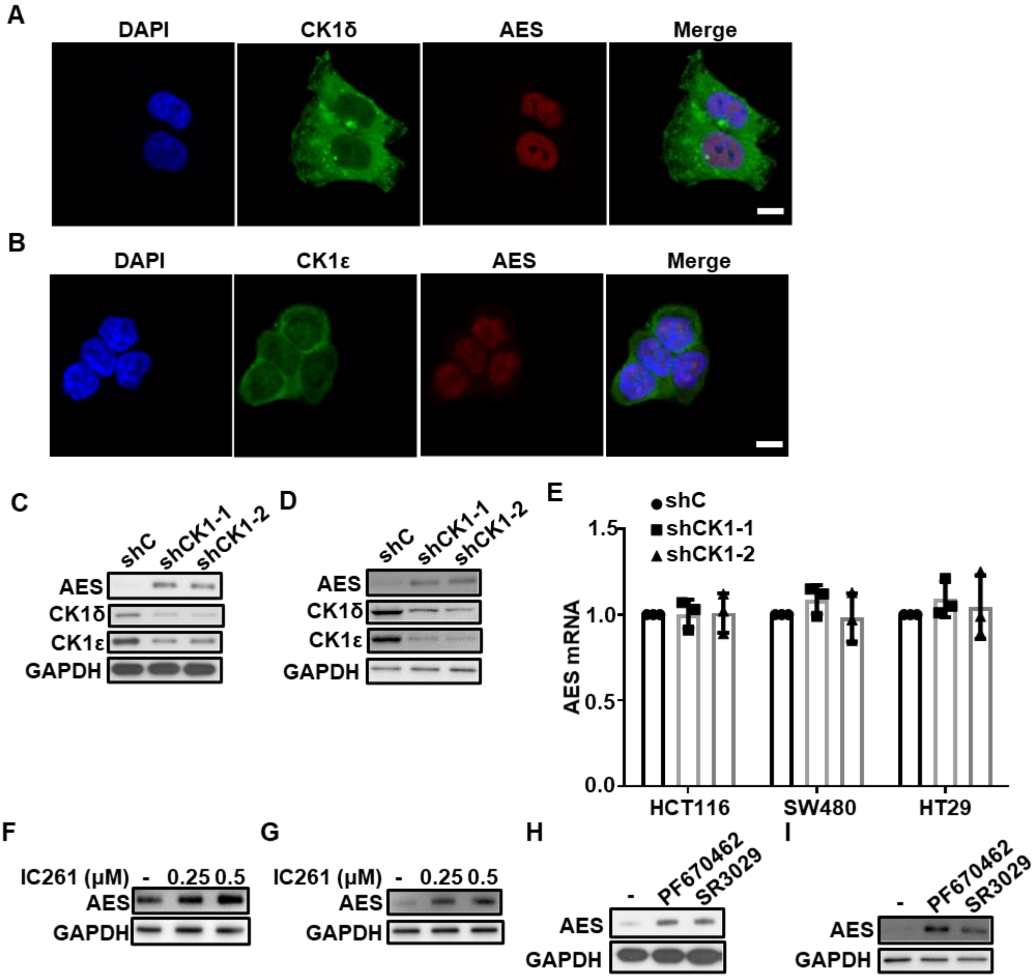

Fig. S1. CK1δ/ε regulates AES stability in CRC cells. (**A**) The colocalization of CK1δ and AES in HCT116 cells was detected by immunofluorescence. (**B**) The colocalization of CK1ε and AES in HCT116 cells was detected by immunofluorescence. Scale bar, 10 μm. (**C** and **D**) SW480 (**C**) or HT29 (**D**) cells were infected with shC, shCK1δ-1/shCK1ε-1 mixture (shCK1-1), or shCK1δ-2/shCK1ε-2 mixture (shCK1-2) lentivirus, then cell lysates were subjected to immunoblotting with the indicated antibodies. (**E**) HCT116, SW480 and HT29 cells were infected with shC, shCK1δ-1/shCK1ε-1 mixture (shCK1-1), or shCK1δ-2/shCK1ε-2 mixture (shCK1-2) lentivirus respectively, and then real-time PCR was used to detect AES mRNA. Quantification of AES mRNA level was normalized to GAPDH. Values are shown as means ± SD (n = 3). (**F**) SW480 cells were treated with DMSO or the indicated amounts of IC261 for 24 h. The protein level of AES was detected by immunoblotting with the indicated

antibodies. (**G**) Similar to (**F**) except that HT29 cells were used. (**H**) Similar to (**F**) except that either PF670462 (2 μM) or SR3029 (100 nM) were used. (**I**) Similar to (**G**) except that either PF670462 (2 μM) or SR3029 (100 nM) were used.

Supplementary Figure S2, Wang et al.

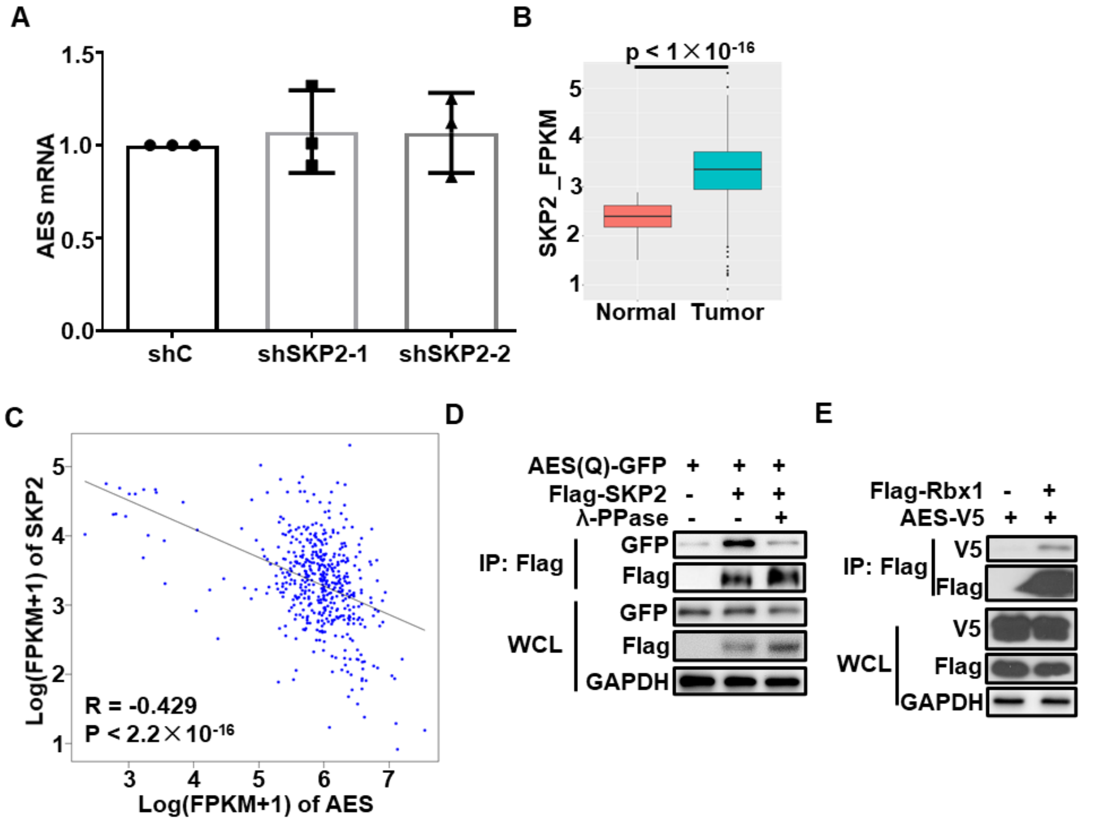

Fig. S2. SKP2 interacts with AES Q domain and promotes the degradation of AES. (**A**) HCT116 cells were infected with shC, shSKP2-1, or shSKP2-2 lentivirus and the mRNA level of AES was detected by real-time PCR. Quantification of AES mRNA level was normalized to GAPDH. Values are shown as means ± SD (n = 3). (**B**) The expression of SKP2 in colon adenocarcinomas and non-malignant adjacent colon tissues using data from the Cancer Genome Atlas (TCGA) database. (**C**) Correlation analysis between SKP2 and AES expression in colon adenocarcinomas using data from TCGA database. (**D**) HEK293T cells were transfected with the indicated expression plasmids and cell lysates were treated without or with λ-PPase before IP with anti-Flag M2 beads. Immunoblot analysis was performed with the indicated antibodies. (**E**) HEK293T cells were transfected with AES-V5 plasmid along with empty vector or

Flag-Rbx1 expression vector, then cell lysates were subjected to IP with anti-Flag M2 beads. Immunoblot analysis was performed using the indicated antibodies.

Supplementary Figure S3, Wang et al.

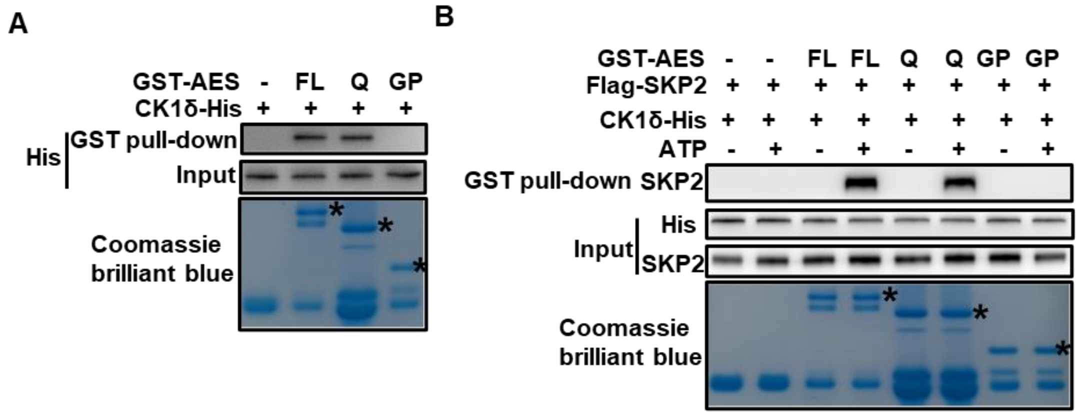

Fig. S3. CK1δ phosphorylates AES to regulate the interaction of SKP2 with AES Q domain. (**A**) *In vitro* GST pulldown assay was performed using purified GST-AES fragments and CK1δ-His from *E. coli*. GST fusion proteins were shown by Coomassie brilliant blue staining and GST was used as a negative control. CK1δ-His was detected by immunoblotting using anti-His antibody. The asterisk represents the GST fusion proteins. (**B**) *In vitro* GST pulldown assay was performed. Purified GST-AES fragments and CK1δ-His were purified from *E. coli.* Flag-SKP2 was immunoprecipitated from HEK293T cells transfected with Flag-SKP2 expression vector. ATP (1 mM) was added as indicated. GST proteins were shown by Coomassie brilliant blue staining and GST was used as a negative control. CK1δ-His and Flag-SKP2 was detected by immunoblotting using anti-His-Tag and anti-SKP2 antibodies, respectively. The asterisk represents the GST fusion proteins.

Supplementary Figure S4, Wang et al.

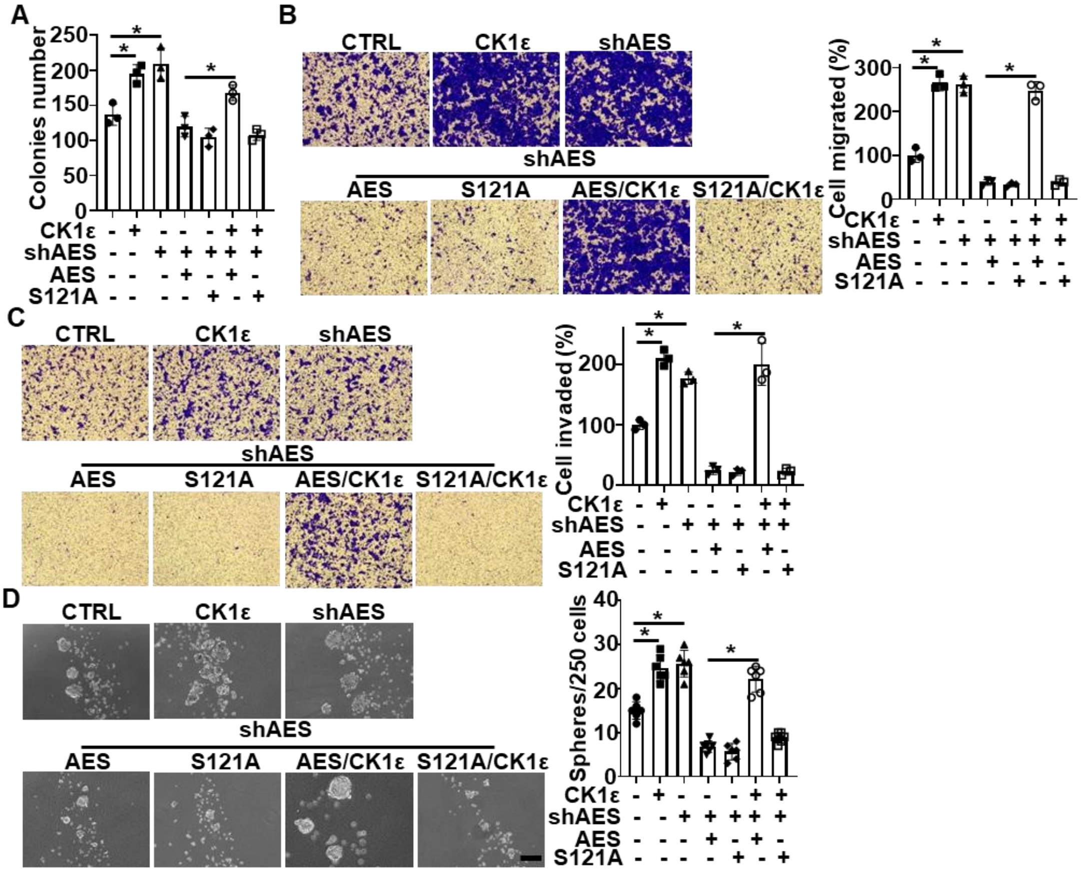

Fig. S4. CK1ε antagonizes the effect of AES on anchorage-independent growth, migration, invasion, and sphere formation in CRC cells. HCT116 cells were infected with the indicated lentivirus. (**A**) Soft agar colony formation assay was performed to evaluate the anchorage-independent growth. Graphical representation of quantitative data showed the relative number of colonies formed (n = 3). (**B** and **C**) Transwell assay was performed to evaluate cell migration (**B**) and invasion (**C**). Cells that migrated or invaded cells through transwells were stained and photomicrographed. Right panel: graphical representation of quantitative data showed the relative number of migrated (**B**) or invaded (**C**) cells (n = 3). (**D**) The sphere-forming ability was assessed in HCT116 cells. Representative images of sphere formation were presented. Right panel: graphical representation of quantitative data showed the relative number of spheres formed (n = 6). Scale bar, 200 μm. Values are shown as means ± SD. *$P < 0.05$; Student's t-test.

Supplementary Figure S5, Wang et al.

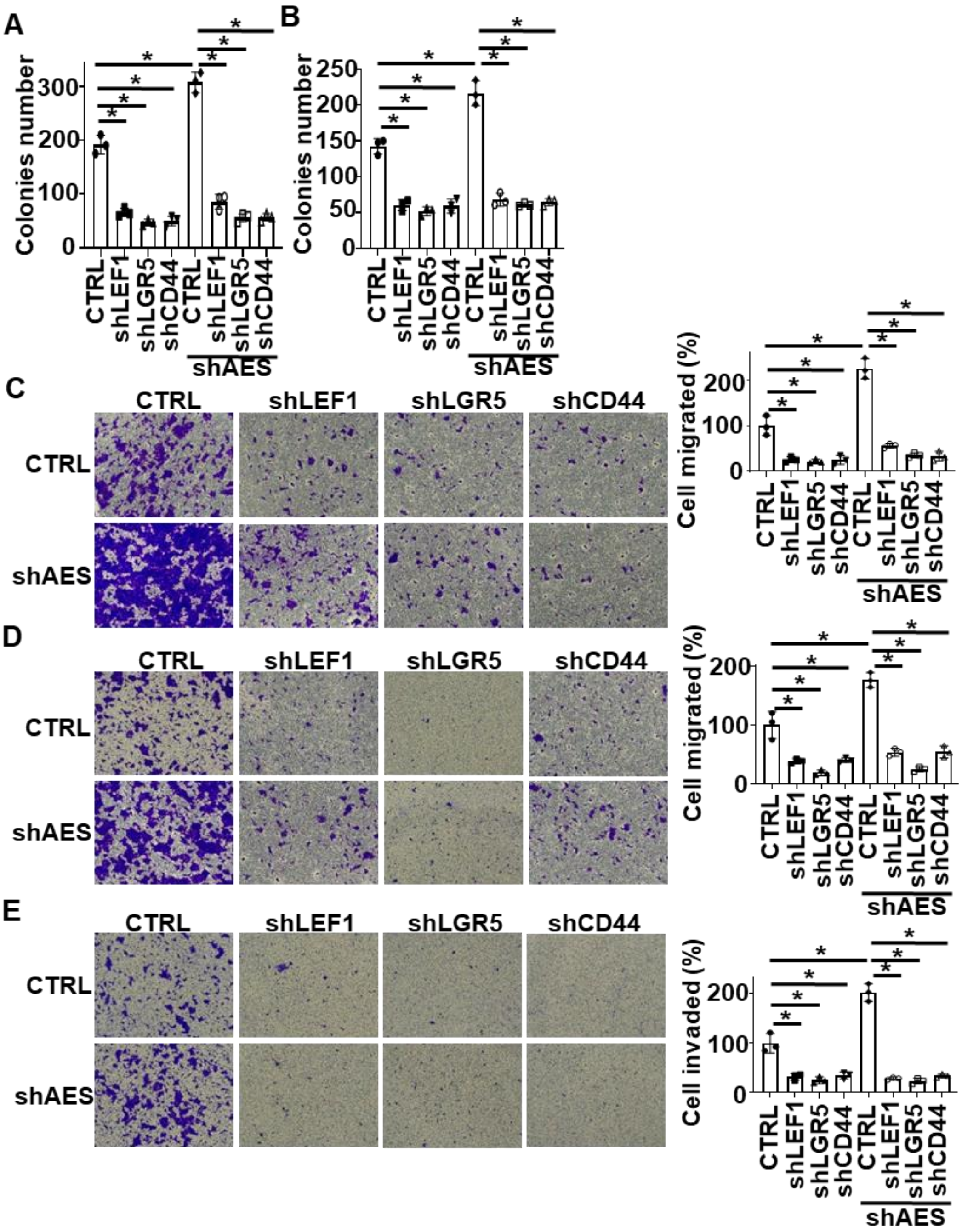

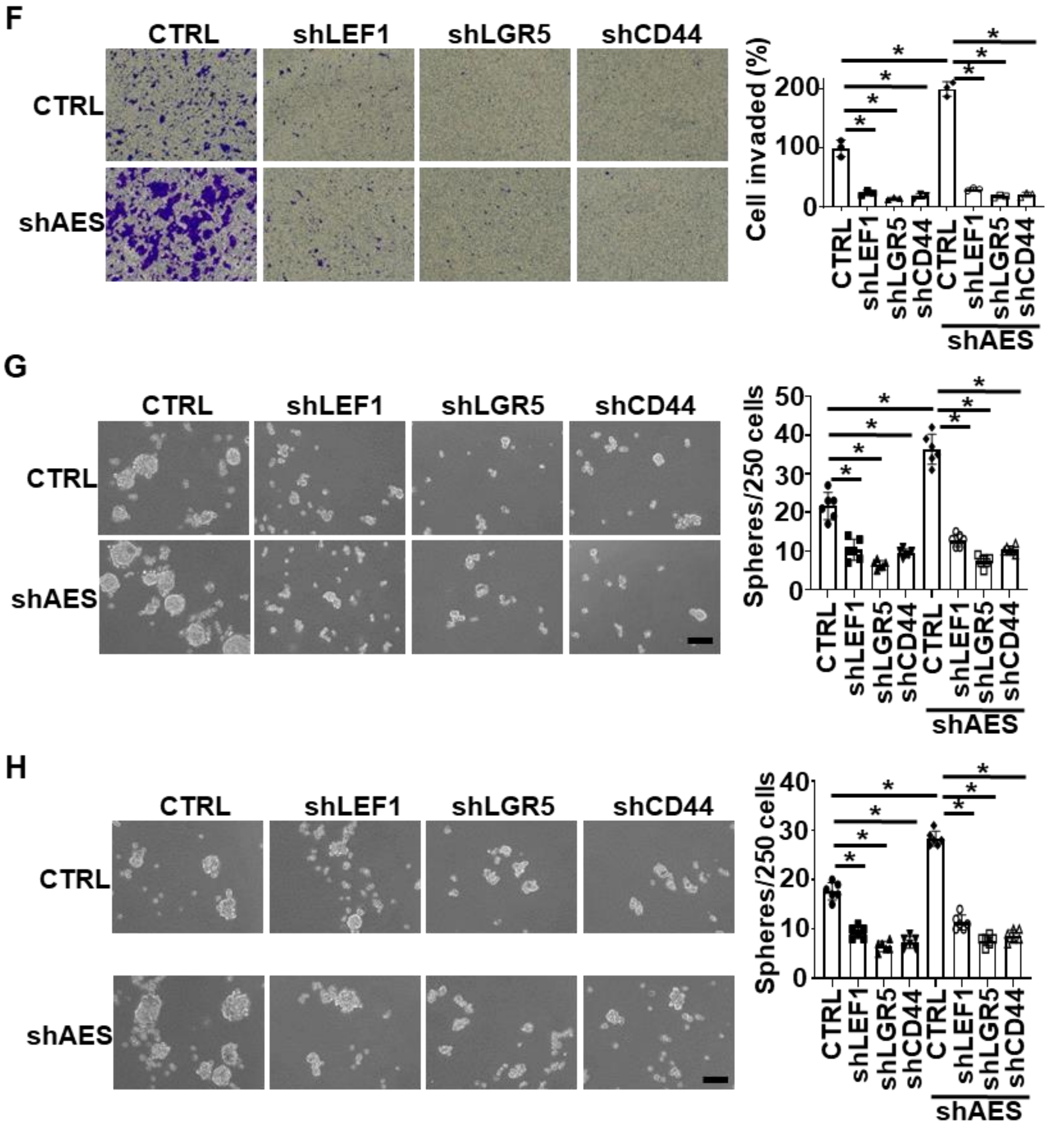

Fig. S5. Some Wnt target genes antagonizes the effect of AES on anchorage-independent growth, migration, invasion, and sphere formation in CRC cells. HCT116 (**A, C, E, G**) or HT29 (**B, D, F, H**) cells were infected with the indicated lentivirus. (**A** and **B**) Soft agar colony formation assay was performed to evaluate the anchorage-independent growth of HCT116 (**A**) or HT29 (**B**) cells. Graphical representation of quantitative data showed the relative number of colonies formed (n = 3). (**C** and **D**) Transwell assay was performed to evaluate cell migration of HCT116 (**C**) or HT29 (**D**) cells. Cells that migrated cells through transwells were stained and photomicrographed. Right panel: graphical representation of quantitative data showed the relative number of migrated cells (n = 3). (**E** and **F**) Transwell assay was performed to evaluate cell invasion of HCT116 (**E**) or HT29 (**F**) cells. Cells that invaded cells

through transwells were stained and photomicrographed. Right panel: graphical representation of quantitative data showed the relative number of migrated cells (n = 3). (**G** and **H**) The sphere-forming ability was assessed in HCT116 (**G**) or HT29 (**H**) cells. Representative images of sphere formation were presented. Right panel: graphical representation of quantitative data showed the relative number of spheres formed (n = 6). Scale bar, 200 μm. Values are shown as means ± SD. *P < 0.05; Student's t-test.

Supplementary Figure S6, Wang et al.

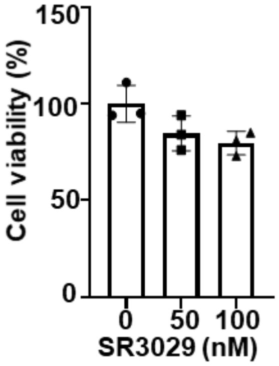

Fig. S6. SR3029 has little effect on cell viability of APC$^{min/+}$ colorectal tumor organoids. Cell viability of APC$^{min/+}$ colorectal tumor organoids was detected by the CellTiter-Glo 3D Cell Viability Assay Kit after treatment with the indicated concentrations of SR3029 for 24 h.

Supplementary Figure S7, Wang et al.

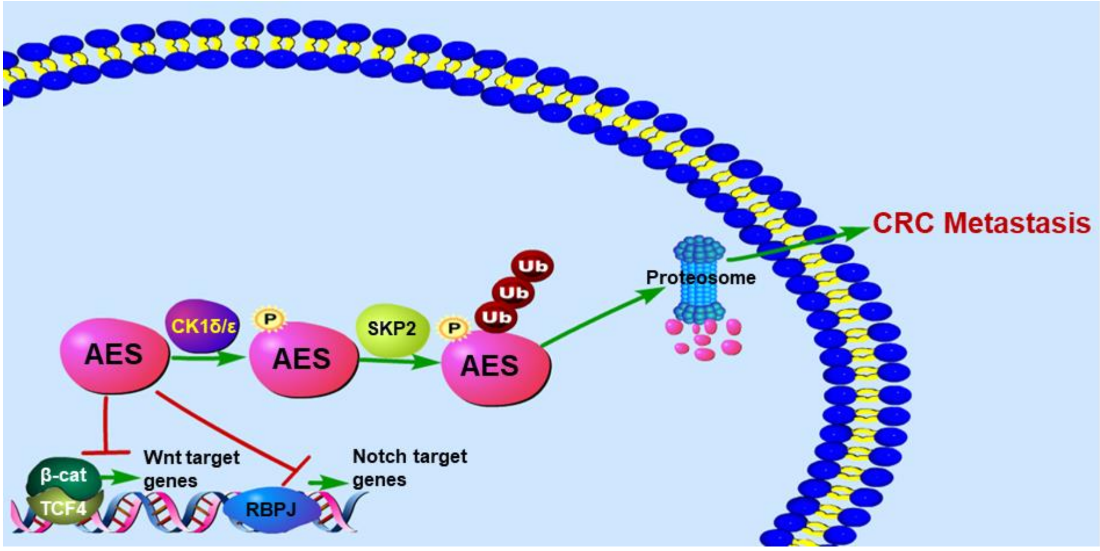

Fig. S7. A proposed model for the regulation of AES stability by CK1δ/ε. AES can be phosphorylated at Ser121 by CK1δ/ε, which facilitates the polyubiquitination and degradation of AES through SKP2-mediated proteasome pathway, and consequently promotes the expression of Wnt and Notch target genes and CRC metastasis.

Supplementary Table 1, Wang et al.

Table S1 Primer sequences used for real-time PCR amplification.

GENE		SEQUENCE (5´->3´)
human AES	**S**	TCCTACGGCTTGAACATCGAG
	**AS**	CTTGGCCCTCTCAATGGCTC
human Axin2	**S**	TACACTCCTTATTGGGCGATCA
	**AS**	TTGGCTACTCGTAAAGTTTTGGT
human Fibronectin	**S**	ACCTACGGATGACTCGTGCTTT
	**AS**	TTCAGACATTCGTTCCCACTCA
human LEF1	**S**	AGGAACATCCCCACACTGAC
	**AS**	AGGTCTTTTTGGCTCCTGCT
human HES1	**S**	CCTGTCATCCCCGTCTACAC
	**AS**	CACATGGAGTCCGCCGTAA
human HES2	**S**	CCAACTGCTCGAAGCTAGAGA
	**AS**	AGCGCACGGTCATTTCCAG
human CD44	**S**	CTGCCGCTTTGCAGGTGTA
	**AS**	CATTGTGGGCAAGGTGCTATT
human LGR5	**S**	CTCCCAGGTCTGGTGTGTTG
	**AS**	GAGGTCTAGGTAGGAGGTGAAG
human GAPDH	**S**	CCAGAACATCATCCCTGCCTCTACT
	**AS**	GGTTTTTCTAGACGGCAGGTCAGGT

Supplementary Table 2, Wang et al.

Table S2 Antibodies used in this study.

Antibodies	Source	Catalog No.	Application
Anti-Flag antibody	Sigma-Aldrich	F1804	WB
Anti-Flag antibody	Cell Signaling Technology	14793	WB
Anti-V5 antibody	Cell Signaling Technology	13202	WB
Anti-AES antibody	Abcam	ab137060	WB, IF
Anti-AES antibody	Abcam	ab118881	IHC
Anti-CK1δ antibody	Santa Cruz	sc-55554	WB
Anti-CK1δ antibody	Abcam	ab151793	IHC
Anti-CK1ε antibody	Cell Signaling Technology	12448	WB
Anti-CK1ε antibody	Abcam	Ab70110	IHC
Anti-GAPDH antibody	TransGen Biotech	HC-301	WB
Anti-Ubiquitin antibody	Santa Cruz	sc-271289	WB
Anti-SKP2 antibody	Cell Signaling Technology	2652	WB
Anti-GFP antibody	Proteintech	66002-1-Ig	WB
Anti-phospho-Ser/Thr antibody	ECM Biosciences	PM3801	WB
Anti-β-catenin antibody	Santa Cruz	sc-7963	WB
Anti-TCF4E antibody	Cell Signaling Technology	2569	WB
Anti-LEF1 antibody	Cell Signaling Technology	2230	WB
Anti-CD44 antibody	Cell Signaling Technology	3570	WB, IHC
Anti-LGR5 antibody	Abcam	ab75732	WB, IHC
Anti-PCNA antibody	GeneTex	GTX100539	WB
Anti-Ki-67 antibody	Biolegend	350501	IHC
Anti-Cleaved Caspase-3 antibody	Cell Signaling Technology	9664	WB
Anti-β-Actin antibody	TransGen Biotech	HC-201	WB

Supplementary Table 3, Wang et al.

Table S3 shRNA sequences used in this study.

GENE	SEQUENCE (5´->3´)
human CK1ε-1	GATCCGCAACCTGGTCTACATCATCGTTCAAGAGACGATGATGTAGACCAGGTTGCTTTTTTGGAAA
human CK1ε-2	GATCCGTATATCCACTCCAAGAACTTCAAGAGAGTTCTTGGAGTGGATATACTTTTTTGGAAA
human CK1δ-1	GATCCGGTCTAGGATCGAAATGTTTTCAAGAGAAACATTTCGATCCTAGACCTTTTTTGGAAA
human CK1δ-2	GATCCGTGATCAGTCGCATCGAATATTCAAGAGATATTCGATGCGACTGATCATTTTTTGGAAA
human AES-1	CCGGGCTGAACTCTATCATCCGACACTCGAGTGTCGGATGATAGAGTTCAGCTTTTT
human AES-2	CCGGTGATGGCGAGAAGTCGGATTACTCGAGTAATCCGACTTCTCGCCATCATTTTT
human SKP2-1	CCGGAAGGTCTCTGGTGTTTGTAAGCTCGAGCTTACAAACACCAGAGACCTTTTTTTG
human SKP2-2	CCGGAAGCATGTACAGGTGGCTGTTCTCGAGAACAGCCACCTGTACATGCTTTTTTTG
human LEF1	CCGGCCATCAGATGTCAACTCCAAACTCGAGTTTGGAGTTGACATCTGATGGTTTTTG
human LGR5	CCGGTAGCCTCCGATCGCTGAATTTCTCGAGAAATTCAGCGATCGGAGGCTATTTTTG
human CD44	CCGGCCGTTGGAAACATAACCATTACTCGAGTAATGGTTATGTTTCCAACGGTTTTTG
mouse CK1ε	CCGGCGAGTTCTCAACATACCTCAACTCGAGTTGAGGTATGTTGAGAACTCGTTTTTG
mouse CK1δ	CCGGTCGTATTGAGTACATTCATTCCTCGAGGAATGAATGTACTCAATACGATTTTTG
mouse AES	CCGGTGACGGAGAGAAGTCGGATTACTCGAGTAATCCGACTTCTCTCCGTCATTTTTG
